# A CRISPR Screen Using Subtilase Cytotoxin Identifies SLC39A9 as a Glycan-Regulating Factor

**DOI:** 10.1016/j.isci.2019.05.005

**Published:** 2019-05-08

**Authors:** Toshiyuki Yamaji, Hisatoshi Hanamatsu, Tsuyoshi Sekizuka, Makoto Kuroda, Norimasa Iwasaki, Makoto Ohnishi, Jun-ichi Furukawa, Kinnosuke Yahiro, Kentaro Hanada

**Affiliations:** 1 Department of Biochemistry and Cell Biology, National Institute of Infectious Diseases, Shinjuku-ku, Tokyo 162-8640, Japan; 2 Department of Advanced Clinical Glycobiology, Faculty of Medicine and Graduate School of Medicine, Hokkaido University, Sapporo 001-0021, Japan; 3 Department of Gastroenterology and Hepatology, Graduate School of Medicine, Hokkaido University, Sapporo 060-8638, Japan; 4 Pathogen Genomics Center, National Institute of Infectious Diseases, Shinjuku-ku, Tokyo 162-8640, Japan; 5 Department of Orthopaedic Surgery, Graduate School of Medicine, Hokkaido University, Sapporo 060-8638, Japan; 6 Department of Bacteriology I, National Institute of Infectious Diseases, Shinjuku-ku, Tokyo 162-8640, Japan; 7 Department of Molecular Infectiology, Graduate School of Medicine, Chiba University, Chiba 260-8670, Japan

**Keywords:** Biological Sciences, Biochemistry, Molecular Biology, Microbiology, Cell Biology

## Abstract

Subtilase cytotoxin (SubAB) is a virulence factor produced by locus of enterocyte effacement-negative Shiga-toxigenic *Escherichia coli* strains. The toxin recognizes sialoglycans for entry and cleaves an endoplasmic reticulum chaperon, binding immunoglobulin protein, to cause cell death. However, no systematic screening has yet been performed to identify critical host factors. Here, we performed a genome-wide CRISPR/Cas9 knockout screen for SubAB-induced cell death and identified various sialoglycan-related and membrane-trafficking genes. Analysis of glycan-deficient cells demonstrated that not only N-glycans but also O-glycans serve as SubAB receptors. In addition, SLC39A9, which is a predicted zinc transporter, as well as KDELRs and JTB, were required for SubAB to induce maximal cell death. Disruption of the *SLC39A9* gene markedly reduced both complex-type N-glycans and core 1 O-glycans, and the O-glycan reduction was attributed to the reduction of core 1 synthase (C1GalT1). These results provide insights into the post-transcriptional regulation of glycosyltransferases by SLC39A9, as well as sialoglycan species as SubAB receptors.

## Highlights

• CRISPR knockout screening identified host factors for SubAB-induced cell death
• O-glycans as well as N-glycans serve as SubAB receptors
• Loss of SLC39A9 as well as loss of KDELR2 and JTB reduces sensitivity to SubAB
• SLC39A9 is required for biosynthesis of complex-type N-glycans and core 1 O-glycans

## Graphical Abstract

**Figure:**
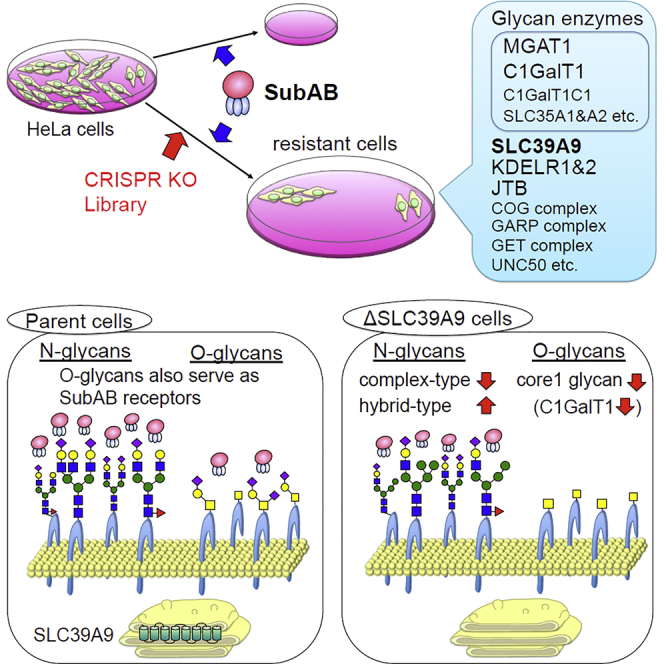

## Introduction

Shiga-toxigenic *Escherichia coli* (STEC) causes various gastrointestinal symptoms in humans, including severe bloody diarrhea, hemorrhagic colitis, and life-threatening hemolytic-uremic syndrome (HUS) (Kaper et al., 2004). Shiga-like toxins (STx1 and 2) are major virulence factors of STEC, whereas some locus of enterocyte effacement (LEE)-negative STEC strains also produce another toxin, subtilase cytotoxin (SubAB), which was discovered in a highly virulent STEC O113:H21 strain, 98NK2 (Paton et al., 2004). SubAB is lethal to mice, causing microvascular damage and HUS-like symptoms (Wang et al., 2007, Wang et al., 2011, Furukawa et al., 2011), indicating that the toxin increases the virulence of STEC.

SubAB utilizes glycans that terminate in sialic acids (SAs) (sialoglycans) as receptors (Byres et al., 2008). After binding to the cell surface, the toxin is retrogradely transported to the endoplasmic reticulum (ER) through the Golgi apparatus; the transport is dependent on the conserved oligomeric Golgi (COG) complex (Zolov and Lupashin, 2005, Smith et al., 2009). Then SubAB cleaves the ER chaperon protein, binding immunoglobulin protein (BiP) (also known as GRP-78), via its subtilase-like serine protease activity (Paton et al., 2004). The cleavage of BiP causes ER stress, which results in cell death (Paton et al., 2006). There have been several detailed reports about SubAB receptors. First, glycans terminating in non-human-derived SA N-glycolylneuraminic acid (Neu5Gc) are the preferred receptors for SubAB, compared with those terminating in N-acetylneuraminic acid (Neu5Ac), which is more commonly observed (Byres et al., 2008). Second, glycosphingolipids (GSLs) containing SA (gangliosides) do not act as receptors for SubAB, which has been demonstrated using ganglioside-deficient mice (Kondo et al., 2009). Third, SubAB binds to several glycoproteins, including integrin and L1 cell adhesion molecule (L1CAM) (Yahiro et al., 2006, Yahiro et al., 2011). However, it is still unclear which type of glycan is actually used by SubAB as a functional receptor in cells and which host factors, including glycan-regulating factors, are critical for SubAB to kill cells.

Clustered regulatory interspaced short palindromic repeat (CRISPR) libraries have been utilized to comprehensively investigate critical factors necessary for toxin action, as well as for virus infection (Shalem et al., 2014, Wang et al., 2014, Blondel et al., 2016, Savidis et al., 2016, Tao et al., 2016, Virreira Winter et al., 2016, Han et al., 2018, Pacheco et al., 2018, Tian et al., 2018). Recently, we performed a genome-wide CRISPR/Cas9 knockout (KO) screen using STx-induced cytotoxicity and identified various genes required for STx receptor and membrane-trafficking functionality, including sphingolipid-related genes (Yamaji et al., 2019).

In this study, we performed a CRISPR KO screen to search for genes that inhibited SubAB-induced cell death when knocked out and identified a number of sialoglycan-related genes as well as membrane trafficking genes. We focused on genes that affected sialoglycan receptors and revealed that not only N-glycans but also O-glycans of glycoproteins serve as SubAB receptors. Furthermore, SLC39A9, a predicted zinc transporter protein, was required for the proper biosynthesis of both N- and O-glycans.

## Results

### Identification of Genes Conferring Resistance to SubAB-Induced Cell Death

**Figure 1:**
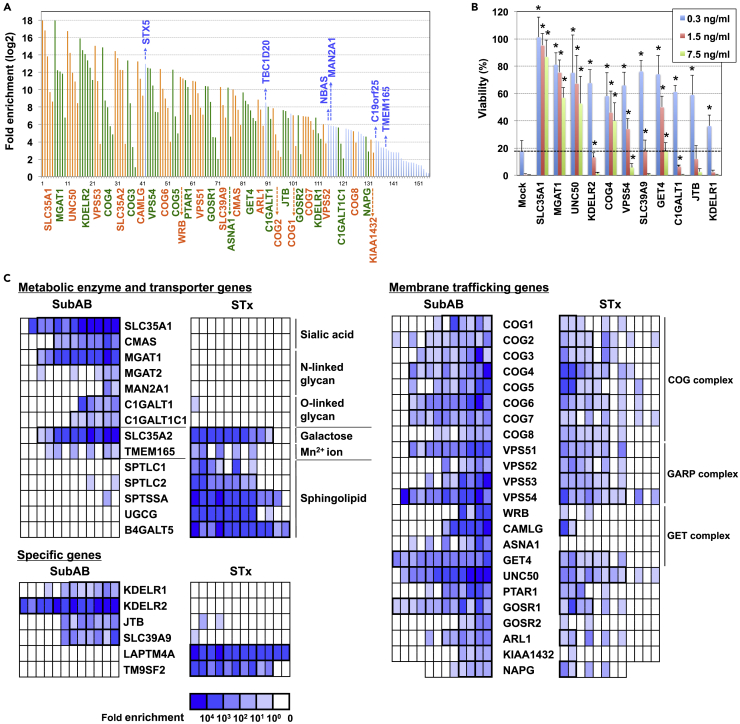
Identification of SubAB Resistance Genes in a Genome-wide CRISPR Screen (A) Identification of sgRNAs enriched in the screen. Fold enrichment represents the average of two independent experiments. Genes, including at least one sgRNA enriched in duplicate, are aligned in descending order of fold enrichment. Orange and green bars indicate that multiple sgRNAs were enriched for a gene, whereas blue bars indicate that a single sgRNA was enriched for a gene. The full raw dataset is shown in Data S1, S2, and S3. (B) Reproducibility of SubAB resistance conferred by individual sgRNAs. Each sgRNA was transduced into HeLa cells. Untransfected cells were excluded using puromycin selection, and successfully transfected cells were then treated for 24 h with SubAB at the indicated concentration (boxes) and further cultured for 4 days in the absence of the toxin. Viability was estimated using an 3-(4,5-Dimethylthiazoyl-2-yl)-2,5-diphenyltetrazolium bromide (MTT) assay, and is expressed as the percentage of the MTT value (OD570) in the absence of SubAB. The percentages shown are the mean percentages ± SD obtained from four independent experiments. The dotted line indicates the viability of mock-transfected cells treated with 0.3 ng/mL SubAB. The Holm-Bonferroni corrected t test was used for multiple comparisons. Asterisks denote statistical significance. (C) Fold enrichment of six sgRNAs in glycan-related and membrane-trafficking genes enriched in the SubAB screen compared with that of an STx screen (Yamaji et al., 2019). The heatmap is representative of individual sgRNA enrichment (sg1–6) in two independent experiments (groups #1 and #2). See also Figures S1 and S2, and Data S1, S2, and S3.

To identify crucial host factors required for SubAB-induced cell death in HeLa cells, we performed a genome-wide CRISPR/Cas9 KO screen. We used a GeCKO v2 pooled library targeting a total of 19,050 human genes with six single-guide RNAs (sgRNAs) per gene (Sanjana et al., 2014). sgRNAs enriched by SubAB treatment in independent duplicate sets were selected as SubAB-resistant sgRNA candidates (Figure 1A; the full raw dataset is shown in Data S1, S2, and S3). The candidates included 155 sgRNAs for 68 genes, with 33 genes containing multiple sgRNAs; most candidates were sialoglycan-related genes, which are required for SubAB receptors, and membrane trafficking-related genes. To validate this screen, 11 identified sgRNAs were individually transduced into HeLa cells to observe any effects of these sgRNAs on SubAB-induced cytotoxicity (Figure 1B). All sgRNAs tested conferred resistance to SubAB, which indicated that this screening was functional.

Recently, we performed a genome-wide screen using STx-induced cytotoxicity (Yamaji et al., 2019). SubAB has similar characteristics to STx in that both toxins recognize glycans and are transported from the plasma membrane to the ER (although the receptor for STx is globotriaosylceramide [Gb3], a neutral GSL); therefore, the genes enriched in the SubAB screen were compared with those from the STx screen (Figure 1C). Glycan enzyme genes specifically enriched in the SubAB screen contained SA-related genes including *CMAS* (cytidine 5′-monophosphate [CMP]-SA synthase) and *SLC35A1* (CMP-SA transporter). Interestingly, as a backbone of sialoglycans, not only N-glycan genes including *MGAT1* (N-acetylglucosaminyl [GlcNAc] transferase I) but also O-glycan genes including *C1GalT1* (core 1 β1,3-galactosyltransferase 1) and its chaperon *C1GalT1C1* (also known as Cosmc) were enriched in the SubAB screen. On the other hand, GSL genes, which were enriched in the STx screen, were not enriched in the SubAB screen. Uridine diphosphate (UDP)-galactose (Gal) transporter (*SLC35A2*) and transmembrane protein 165 (*TMEM165*), a Mn^2+^ transporter required for some glycosyltransferases (Potelle et al., 2016), were commonly observed in both the SubAB and STx screens, reflecting that UDP-Gal and Mn^2+^ are required for the synthesis of both glycan receptors.

Besides the glycan enzyme genes, four genes were specifically enriched in the SubAB screen including KDEL receptors 1 and 2 (*KDELR1* and *2*), jumping translocation breakpoint (*JTB*), and *SLC39A9*, which encodes a predicted zinc transporter protein. Conversely, *LAPTM4A* and *TM9SF2*, which were enriched in the STx screen and identified as factors involved in the regulation of GSL synthesis by three independent groups (Yamaji et al., 2019, Pacheco et al., 2018, Tian et al., 2018), were not enriched in the SubAB screen. In addition to these specific genes, many membrane trafficking genes were commonly enriched in both screens, including the COG complex (*COG1-8*), involved in intra-Golgi retrograde transport (Blackburn and Lupashin, 2016); the GARP complex (*VPS51-54*) and *UNC50*, involved in retrograde transport from endosomes to the *trans-*Golgi network (Bonifacino and Hierro, 2011, Selyunin et al., 2017); the GET complex (*GET4, CAMLG*), involved in ER translocation of tail-anchored membrane proteins (Stefanovic and Hegde, 2007); and other membrane trafficking genes (*PTAR1*, *GOSR1*, *ARL1*, and *NAPG*). These genes participate in general glycan synthesis or retrograde transport of both toxins.

Ricin is a plant toxin that binds to glycan ligands with terminal galactose for cell entry. A previous CRISPR screen using ricin-induced cytotoxicity identified various glycan-related genes and membrane trafficking genes (Tian et al., 2018); we therefore compared our SubAB screen with this ricin screen (Figure S1). Several N-glycan-related genes, including *MGAT1* and membrane trafficking genes such as the GARP complex, were commonly enriched in the two screens. *JTB* enrichment was also observed in both screens, whereas *KDELRs*, *SLC39A9*, and O-glycan-related genes were not enriched in the ricin screen. We hereafter focused on two questions. First, to what level do N- and O-glycans serve as receptors for SubAB? Second, how do KDELRs, JTB, and SLC39A9 affect SubAB-induced cell death?

### Construction of *MGAT1* and *C1GalT1* and Their Double KO HeLa Cells

To address to what level N- and O-glycans function as SubAB receptors, *MGAT1* KO (ΔMGAT1), *C1GalT1* KO (ΔC1GalT1), and double KO (ΔC1GalT1/ΔMGAT1 DKO) cell clones were generated using the CRISPR/Cas9 system. Sequence analyses demonstrated that the coding regions of the respective genes were frame shifted in all alleles (Figure S2A). Quantitative N- and O-glycan analyses were performed using matrix-assisted laser desorption/ionization-time of flight (MALDI-TOF) mass spectrometry to verify glycan alterations in these KO cells, according to previously described procedures: a glycoblotting method for N-glycan analysis and a microwave-assisted β-elimination in the presence of pyrazolone analogs method for O-glycan analysis (Furukawa et al., 2008, Furukawa et al., 2015). In ΔMGAT1 and ΔC1GalT1/ΔMGAT1 DKO cells, no complex-/hybrid-type N-glycans were detected. Instead, high-mannose-type N-glycans containing five mannose residues (HM5), which are precursors of complex-/hybrid-type N-glycans, had accumulated, confirming that these KO cells did not express N-linked sialoglycans (Figure S2B, raw quantitative data is shown in Data S4). On the other hand, O-glycan analysis demonstrated that T antigen, which is synthesized by C1GalT1, and the following sialylated and disialylated T antigens were not detected in ΔC1GalT1 and ΔC1GalT1/ΔMGAT1 cells, as we expected (Figure S2C, raw quantitative data are shown in Data S4). Notably, Neu5Gc-containing glycans were hardly detected in HeLa cells at all under our conditions.

### Both ΔMGAT1 and ΔC1GalT1 Cells Are Resistant to SubAB

**Figure 2:**
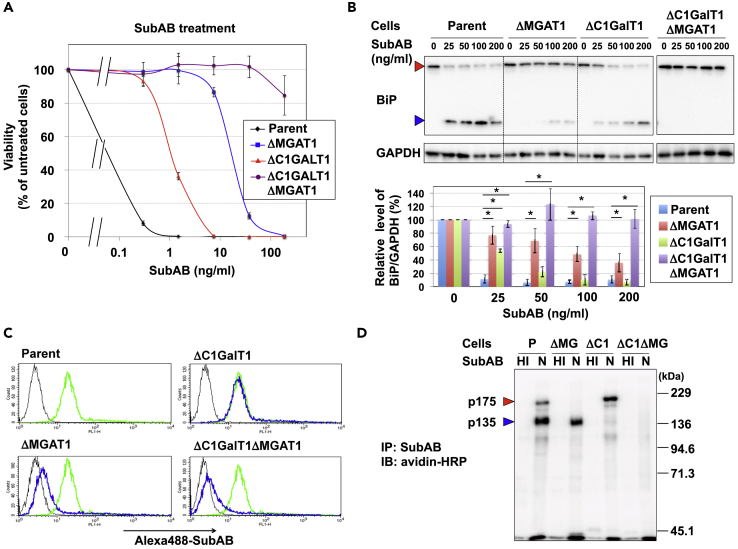
Both N- and O-glycans Serve as SubAB Receptors (A) SubAB sensitivity in glycan-gene KO cells. Cells were treated with SubAB as described in Figure 1B at the indicated concentration. Viability was estimated as described for Figure 1B and is expressed as the mean percentage of the MTT value (OD570) in the absence of SubAB. The percentages shown are the mean percentages ± SD obtained from three independent experiments. (B) BiP cleavage in glycan-gene KO cells. Cells were incubated with SubAB at the indicated concentration of SubAB for 12 h. SubAB-induced BiP cleavage was determined by immunoblots using anti-BiP monoclonal antibodies. GAPDH served as a loading control. Quantification of BiP uncleaved (78 kDa) by SubAB was performed by densitometry. The percentages shown are the mean percentages ± SD obtained from three independent experiments. The Bonferroni-corrected t test was used for multiple comparisons. *p < 0.017. (C) Surface binding of SubAB on glycan-gene KO cells. Cells were stained with (blue lines) or without (black lines) Alexa 488-labeled SubAB (Alexa 488-SubAB) and analyzed using FACS. Yellow-green lines in all panels indicate staining in parent cells. (D) Detection of SubAB-binding proteins in glycan gene KO cells. Biotinylated cell surface proteins prepared from the indicated cells were immunoprecipitated with heat-inactivated (HI) or native (N) SubAB as described in the Transparent Methods section. SubAB-binding proteins were detected with streptavidin-horseradish peroxidase (HRP). Data are representative of at least three separate experiments. See also Figure S2 and Data S4.

Toxin resistance in our established KO cells was tested by exposing the cells to various concentrations of SubAB, and then determining cell viability (Figure 2A). ΔMGAT1 cells were resistant to SubAB in a dose-dependent manner, which is consistent with previous results that suggested that N-glycans are important for SubAB binding to glycoproteins (Yahiro et al., 2006). The ΔC1GalT1 cells also showed resistance to SubAB, although they were less resistant than ΔMGAT1 cells. This difference was consistent with the effect of the early cleavage of BiP following SubAB treatment; BiP reduction was more marked in ΔMGAT1 cells compared with ΔC1GalT1 cells (Figure 2B). Notably, ΔC1GalT1/ΔMGAT1 DKO cells were completely resistant to SubAB even at about 200 ng/mL, a concentration that killed ΔMGAT1 cells (Figure 2A). BiP cleavage by SubAB was also completely inhibited in ΔC1GalT1/ΔMGAT1 cells (Figure 2B). These results suggest that not only N-glycans but also O-glycans affect SubAB-induced BiP cleavage and cell death.

Next, the binding of SubAB to cell surface receptors was examined by two distinct methods: flow cytometry and immunoprecipitation analysis. Flow cytometry demonstrated that binding of fluorescence-labeled SubAB to cell surfaces was reduced in ΔMGAT1 cells and ΔC1GalT1/ΔMGAT1 cells, but not in ΔC1GalT1 cells (Figure 2C). This result indicates that most SubAB binds to N-glycans on the cell surface. Then, to detect detailed interactions of SubAB with cell surface proteins, biotinylated cell surface proteins were pulled down with native (N) or heat-inactivated (HI) SubAB, and the binding proteins were visualized using streptavidin-horseradish peroxidase (Figure 2D). In parent cells, biotinylated p175 and p135 proteins were clearly detected as SubAB-binding proteins in this condition. The interaction between SubAB and p175 disappeared in ΔMGAT1 cells, whereas binding of SubAB to p135 was reduced in ΔC1GalT1 cells. In ΔC1GalT1/ΔMGAT1 cells, fewer SubAB-binding proteins were detected. These results indicate that SubAB binds not only N-glycosylated proteins but also O-glycosylated proteins. Previous studies demonstrated that the 175-kDa band included an L1CAM, and the 135-kDa band included β1 integrin subunit (β1ITG) and hepatocyte growth factor receptor (MET) (Yahiro et al., 2011). The binding of L1CAM and β1ITG to SubAB was N-glycan dependent, because neither L1CAM nor β1ITG were co-immunoprecipitated with SubAB in ΔMGAT1 cells (Figures 2D, S2D, and S2E). The binding of MET to SubAB, however, was likely O-glycan dependent, because knockdown of MET reduced p135, suggesting that MET was the major protein in p135 (Figure S2F), and SubAB-binding biotinylated p135 was reduced in ΔC1GalT1 cells but not in ΔMGAT1 cells (Figures 2D and S2F). It is noteworthy that p135 included O-glycan-modified proteins other than MET, because SubAB-binding proteins were still observed at p135 in MET-knockdown ΔMGAT1 cells (Figure S2F). The reason why N-glycan-dependent but not O-glycan-dependent binding of SubAB was detected by flow cytometry is yet to be determined. It may have been due to lower expression on the cell surface. The results of the immunoprecipitation analysis together with the sensitivity to SubAB suggested that N-glycans of host cells play the predominant role as SubAB receptors, whereas O-glycans also play a minor but significant role as SubAB receptors.

### Disruption of *KDELR2*, *JTB*, and *SLC39A9* Genes Reduces Sensitivity to SubAB

**Figure 3:**
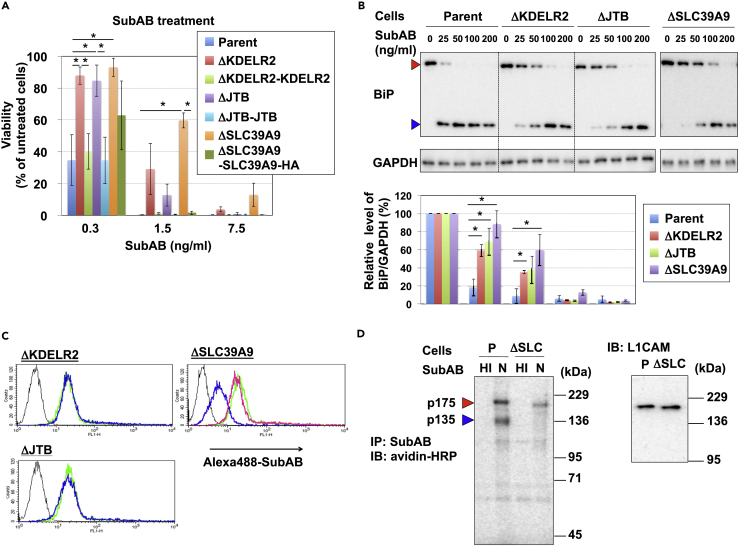
SLC39A9 Regulates the Receptor Expression (A) SubAB sensitivity in *KDELR2*, *JTB*, and *SLC39A9* KO cells and their revertant cells. HA-tagged SLC39A9 was used in SLC39A9 revertant cells. Cells were treated with SubAB at the indicated concentrations. Viability was estimated as described for Figure 1B and is expressed as mean percentages ± SD obtained from three independent experiments. The Bonferroni corrected t test was used for multiple comparisons. *p < 0.0083. (B) BiP cleavage in *KDELR2*, *JTB*, and *SLC39A9* KO cells. Cells were incubated with the indicated concentration of SubAB for 12 h. SubAB-induced BiP cleavage was determined as described for Figure 2B. Experiments were repeated three times with similar results (upper panel). Data are the mean percentages ± SD obtained from three independent experiments. The Bonferroni corrected t test was used for multiple comparisons. *p < 0.017. (C) Surface binding of SubAB on *KDELR2*, *JTB*, and *SLC39A9* KO cells. Cells were stained with (blue lines) or without (black lines) Alexa 488-labeled SubAB (Alexa 488-SubAB) and analyzed using FACS. Yellow-green lines in all panels indicate staining in parent cells. A magenta line indicates staining in cDNA-restored cells. (D) Detection of SubAB-binding proteins in *KDELR2*, *JTB*, and *SLC39A9* KO cells. In the left image, biotinylated cell surface proteins prepared from the indicated cells were immunoprecipitated and detected as described for Figure 2D. Data are representative of at least three separate experiments. In the right image, L1CAM proteins in whole lysates were detected with anti-L1CAM antibodies. See also Figure S3.

To investigate the role of the *KDELR2*, *JTB*, and *SLC39A9* genes in SubAB-induced cell death, we next generated KO cell clones for each gene using the CRISPR/Cas9 system (Figure S3A). These KO cells were resistant to SubAB-induced cell death (Figure 3A) as well as BiP cleavage (Figure 3B). Flow cytometry demonstrated that cell surface binding of SubAB was reduced in ΔSLC39A9 cell, but not altered in ΔKDELR2 and ΔJTB cells (Figure 3C). The reduction of SubAB-binding in ΔSLC39A9 cells was restored by expression of wild-type (WT) SLC39A9, eliminating the possibility of off-target effects. As shown in Figure 2C, binding of SubAB to the cell surface, as detected by flow cytometry, was N-glycan dependent, suggesting that N-glycans at least were altered in ΔSLC39A9 cells. On the other hand, immunoprecipitation analysis of SubAB using biotinylated cell surface proteins showed that O-glycan-dependent (mainly p135) bands were markedly reduced in ΔSLC39A9 cells, as in ΔC1GalT1 cells, but not in other cells (Figures 3D and S3B). These results suggest that SLC39A9 plays a crucial role in N-glycan and O-glycan modifications to cell surface proteins that bind SubAB.

### *SLC39A9* KO Cells Are Defective in N-glycan Biosynthesis

**Figure 4:**
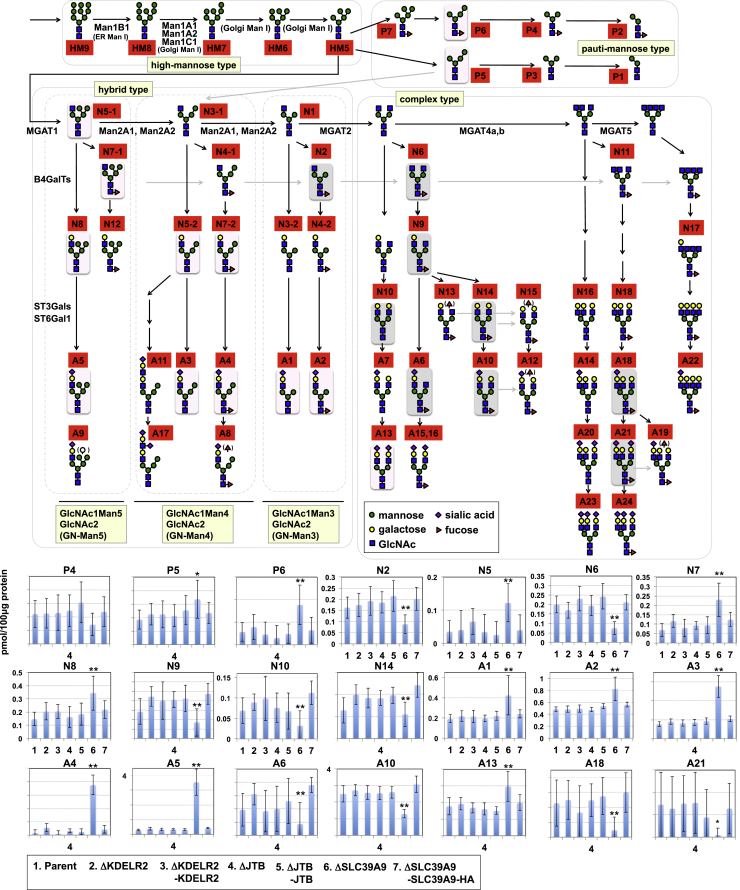
Alteration of N-glycans in *SLC39A9* KO Cells Quantitative analysis of N-glycan species shown in *KDELR2*, *JTB*, and *SLC39A9* KO cells, and their revertant cells. A schematic of N-glycan biosynthesis is shown in the upper section. Red boxes indicate glycan structure numbers as shown in Data S5. Pink boxes indicate statistically increased glycans, and gray boxes indicate statistically decreased glycans in *SLC39A9* KO cells as illustrated in the graphs below. Glycans N3, N4, N5, and N7 may take one of two structures; therefore they are distinguished as N3-1, N3-2, N4-1, N4-2, etc. The graphs show the expression levels of respective N-glycan species compared among the indicated cells (#1–7): mean expression levels ± SD obtained from eight independent experiments. The full raw data set is shown in Data S5. The Bonferroni corrected t test was used for multiple comparisons. *p < 0.025; **p < 0.0083. See also Figures S3 and S4, and Data S5.

First, N-glycans of glycoproteins expressed in ΔSLC39A9 cells were quantitatively analyzed using MALDI-TOF mass spectrometry. As shown in Figure 4, high-mannose N-glycans are processed by various glycosidases and glycosyltransferases at the ER and the Golgi apparatus to form hybrid- and complex-type N-glycans. The Golgi GlcNAc transferases, MGAT1–5, determine the number of GlcNAc branches, followed by galactosylations and sialylations. In this analysis, five high-mannose-type glycans, seven pauti-mannose-type glycans, 18 neutral complex-/hybrid-type glycans, and 24 acidic complex-/hybrid-type glycans were detected (Figures 4 and S3C–S3E, raw quantitative data are shown in Data S5). The total amount of each type of N-glycan was nearly equal among all tested cells (Figure S3C). However, the complex-/hybrid-type N-glycan pattern was markedly different in ΔSLC39A9 compared with parent cells and cDNA-restored cells, with an increased amount of acidic complex-/hybrid-type N-glycans (Figures S3D and S3E), whereas the N-glycan patterns were unchanged in ΔKDELR2 and ΔJTB cells.

The amounts of each N-glycan species were then compared between ΔSLC39A9 and parent cells or the *SLC39A*9 cDNA-restored cells (Figure 4). Hybrid-type GlcNAc1Man4GlcNAc2 (named GN-Man4) and GlcNAc1Man5GlcNAc2 (named GN-Man5) N-glycan groups, containing four or five mannose residues with one GlcNAc residue synthesized by MGAT1, increased in ΔSLC39A9 cells. Acidic glycans (A3–5) in particular were much more frequent than neutral glycans (N5, N7, and N8). Complex-type N-glycans (N6, N10, A10, A18, and A21) and a hybrid-type GlcNAc1Man3GlcNAc2 (named GN-Man3) N-glycan group (N2) were reduced, although some complex- and hybrid-type GN-Man3 sialoglycans (A1, A2, and A13) were a little upregulated. These results suggested two effects on N-glycan synthesis. (1) KO of *SLC39A9* repressed the conversion of GN-Man5 glycans to GN-Man3 glycans, which is normally catalyzed by Golgi α-mannosidase II (MAN2A1 and MAN2A2). (2) KO of *SLC39A9* accelerated sialylation, because increasing levels of acidic glycans (A2 and A13 in the GN-Man3 glycan group, and A3–5 in the GN-Man4 and GN-Man5 glycan groups) were higher than the upstream neutral glycans (N2 and N10 in GN-Man3 glycan groups, and N5, N7, and N8 in GN-Man4 and GN-Man5 glycan groups). As a result, hybrid-type mono-antennary sialoglycans increased to a greater extent and complex-type sialoglycans decreased. Together with Figure 3C, our results suggest that the binding of SubAB to the cell surface of ΔSLC39A9 cells decreased due to N-glycan alterations. Treatment with swainsonine, a Golgi α-mannosidase II inhibitor, also reduced the binding of SubAB to the cell surface (Figure S4).

### *SLC39A9* KO Cells Are Defective in O-glycan Biosynthesis

**Figure 5:**
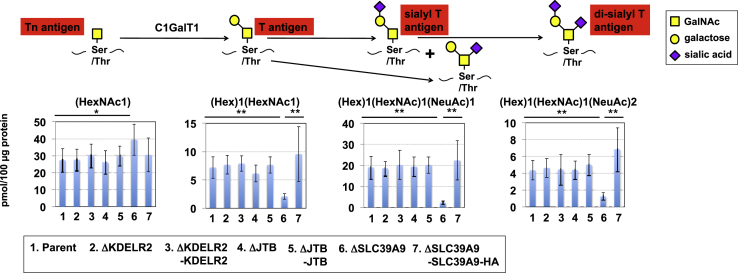
Alteration of O-glycans in *SLC39A9* KO Cells Quantitative analysis of O-glycan species in the indicated cells shown in Figure 4. A schematic of O-glycan biosynthesis is shown in the upper section. Expression levels of respective O-glycan species were compared among the indicated cells (#1–7): mean expression levels SD obtained from eight independent experiments. The full raw dataset is shown in Data S5. The Bonferroni corrected t test was used for multiple comparisons. *p < 0.025; **p < 0.0083. See also Figure S3 and Data S5.

Next, O-glycans of glycoproteins expressed in ΔSLC39A9 cells were analyzed. The major O-glycans in parent cells included four structures: (1) Tn antigen (N-acetyl galactosamine (GalNAc)-Ser/Thr), (2) T antigen (Gal β1,3 GalNAc-Ser/Thr), (3) sialyl T antigen (SA α2,3 Gal β1,3 GalNAc-Ser/Thr), and (4) di-sialyl T antigen (SA α2,3 Gal β1,3 (SA α2,6) GalNAc-Ser/Thr) (the latter three structures are categorized as core 1 O-glycan structures) (Figures 5 and S3F, and Data S5). ΔSLC39A9 cells showed dramatic reductions in the O-glycan T, sialyl T, and di-sialyl T antigens, whereas Tn antigens increased slightly (similar changes were not observed for ΔKDELR2 and ΔJTB cells). Most O-glycans in ΔSLC39A9 cells were therefore Tn antigens (Figure S3F), which is similar to the O-glycan pattern in ΔC1GalT1 cells shown in Figure S2C. These results indicate that SLC39A9 is required for core 1 O-glycan biosynthesis, and the marked reduction of core-1 O-glycans in ΔSLC39A9 cells should contribute to reduced sensitivity to SubAB, because ΔC1GalT1 cells showed a similar reduced sensitivity.

### The Expression Level of C1GalT1 Was Reduced in ΔSLC39A9 Cells

**Figure 6:**
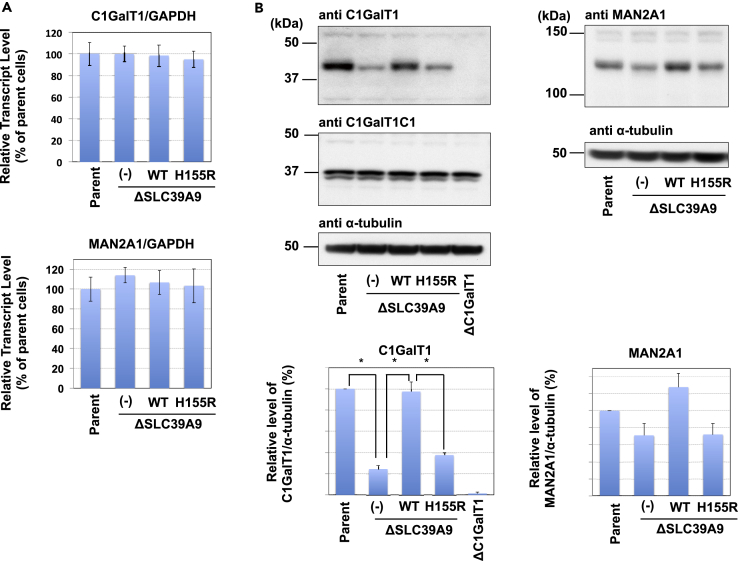
Loss of SLC39A9 Reduces C1GalT1 Proteins (A) Quantitative real-time PCR of C1GalT1 and MAN2A1 mRNAs in parent cells, *SLC39A9* KO cells, wild-type SLC39A9-expressing *SLC39A9* KO cells, and H155R mutant-expressing *SLC39A9* KO cells. Relative mRNA levels of C1GalT1 and MAN2A1 are expressed as the percentage of the value in parent cells and represent the mean percentages ± SD obtained from three independent experiments. (B) Western blot analysis for C1GalT1, C1GalT1C1, and MAN2A1 proteins in parent cells, *SLC39A9* KO cells, wild-type SLC39A9-expressing *SLC39A9* KO cells, and H155R mutant-expressing *SLC39A9* KO cells. The amounts of proteins were expressed as a percentage of the intensity of bands in parent cells: mean percentages ± SD. obtained from three independent experiments. The Bonferroni corrected *t* test was used for multiple comparisons. *p < 0.017. See also Figure S5.

As described above, the loss of SLC39A9 reduced core 1 O-glycans (whose synthesis requires C1GalT1) as well as GN-Man3-hybrid- and the following complex-type N-glycans (whose synthesis requires MAN2A1). Transcriptional levels of C1GalT1 and MAN2A1 did not change (Figure 6A); therefore, we next investigated the protein levels of C1GalT1 and MAN2A1 (Figure 6B). KO of the *SLC39A9* gene markedly reduced C1GalT1 expression, and this reduction was recovered by the expression of WT SLC39A9, suggesting that reduced C1GalT1 is the cause of decreased core 1 O-glycans in ΔSLC39A9 cells. C1GalT1C1, also enriched in this SubAB-resistant screen (Figure 1A), is an ER chaperon required for the expression and activity of C1GalT1 (Ju and Cummings, 2002); therefore there is a possibility that the reduction of C1GalT1 in Δ*SLC39A9* cells was due to a reduction of this chaperon. However, the expression level of C1GalT1C1 was not altered in Δ*SLC39A9* cells. MAN2A1 expression was slightly decreased in Δ*SLC39A9* cells, although this was not statistically significant. The cause of the N-glycan deficiency in Δ*SLC39A9* cells was unlikely to have been due to the small decrease in MAN2A1.

### Histidine 155 of SLC39A9 Is Required for Glycan-Regulating Activity

SLC39A9 is one of 14 Zrt- and Irt-like protein (ZIP) family proteins in humans and is predicted to be a zinc transporter. From X-ray crystallography of a ZIP family member and three-dimensional modeling of rat SLC39A9 (Zhang et al., 2017, Bulldan et al., 2017), a highly conserved amino acid residue, His-155 (H155), was predicted to be an essential amino acid for Zn^2+^ binding in human SLC39A9. Furthermore, replacement of the conserved histidine with arginine abolished Zn^2+^ transport activity in human SLC39A4 (Zhang et al., 2017). To determine the indispensability of H155 for the glycan-regulating activity of human SLC39A9, we constructed an arginine replacement mutant, SLC39A9 (H155R) (Figure S5A). Hemagglutinin (HA)-tagged SLC39A9 WT and mutant SLC39A9 (H155R) were expressed in ΔSLC39A9 cells, and the expression of SLC39A9 WT and H155R was confirmed by western blot using anti-SLC39A9 or anti-HA antibodies (Figure S5B). SLC39A9 WT was co-localized with GM130, a Golgi marker protein (Figure S5C), which is consistent with a previous report (Matsuura et al., 2009). The H155R mutation did not affect the Golgi localization of SLC39A9 (Figure S5C). However, the H155R variant did not recover SubAB binding on the cell surface as well as the expression level of C1GalT1 and MAN2A1 in ΔSLC39A9 cells (Figures 6B and S5D). These results indicated that H155 is indispensable if SLC39A9 is to exhibit its glycan-regulating activity.

## Discussion

In this study, we performed a genome-wide CRISPR library screening for SubAB-induced cell death and found that various genes (e.g., sialoglycan-related genes and membrane trafficking-related genes) were comprehensively enriched in SubAB-resistant cells. Further analyses elucidated two insights. (1) Not only N-glycans but also O-glycans serve as receptors for SubAB and (2) SLC39A9 regulates both N- and O-glycan biosynthesis and loss of SLC39A9 decreased C1GalT1.

Sialoglycans are required for binding of SubAB to the cell surface, which is consistent with our screening result that *CMAS* and *SLC35A1* were highly enriched in this screen. Neu5Gc-containing sialoglycans are believed to be a preferential receptor for SubAB compared with Neu5Ac-containing sialoglycans (Byres et al., 2008). However, Neu5Gc-containing glycans were barely detected in this study, therefore Neu5Gc-dependent enhancement of SubAB binding was not investigated. In this screen, N-glycan-related genes (e.g., *MGAT1*) and O-glycan-related genes (e.g., *C1GalT1*) were enriched (but no GSL-related genes, e.g., *UGCG*, were enriched), and cells with both *MGAT1* and *C1GalT1* genes knocked out conferred complete resistance to SubAB; these findings indicate that glycoproteins serve as SubAB receptors. The crystal structure of the SubB pentamer showed that the binding pockets for receptors are located on the side of SubB (Byres et al., 2008), although receptor recognition domains of other AB5 toxins (e.g., STx, cholera toxin) are located on the bottom of B subunits. These findings suggest that SubAB receptors require some extension of length from cell surface for glycan ligands to access the pocket. Ricin is another toxin that binds to glycoproteins as its receptors; however, in a genome-wide CRISPR screen of ricin-induced cytotoxicity (Tian et al., 2018), O-glycan-related genes were not enriched (Figure S1). Therefore this O-glycan dependence may be a specific feature of SubAB. SubAB binds to several glycoproteins including L1CAM (p170), β1ITG (p135), and MET (p135); most of these interactions are N-glycan dependent (Yahiro et al., 2011). We confirmed that N-glycans play a prominent role as SubAB receptors. O-glycans also serve as receptors, with MET likely to be an O-glycan-dependent receptor, a finding that has not been shown before. In this screen, no L1CAM, β1ITG, or MET were enriched, suggesting that no specific glycoproteins are responsible for being SubAB receptors, but rather various proteins can act as receptors if they have sialoglycans.

In N-glycans of ΔSLC39A9 cells, hybrid-type mono-antennary sialoglycans were markedly increased and complex-type sialoglycans were reduced. Although the total amount of sialoglycans was somewhat increased in ΔSLC39A9 cells (Figure S3E), the binding of SubAB to N-glycans was reduced according to fluorescence-activated cell sorting (FACS) analysis, which specifically detected N-glycan-dependent binding of SubAB (Figures 2C and 3C). The relationship between the number of antennae in N-glycans and their SubAB binding affinity has not been investigated, but bi-antennary or multi-antennary sialoglycans may function as more effective SubAB receptors compared with mono-antennary sialoglycans. All hybrid-type GN-Man5 and GN-Man4 glycans increased, whereas neutral hybrid-type GN-Man3 glycans and complex-type glycans decreased in ΔSLC39A9 cells. MAN2A1 and its homolog, MAN2A2, catalyze the trimming of GN-Man5 to GN-Man3 glycans; a study using *MAN2A1/MAN2A2* DKO mouse embryos showed a complete lack of complex-type glycans, with only hybrid-type GN-Man5 (but not GN-Man4) glycans accumulating (Hato et al., 2006). However, hybrid-type GN-Man4 glycans did accumulate in *MAN2A1* single KO mouse embryos, which is similar to the glycan pattern seen in ΔSLC39A9 cells. Therefore we think that MAN2A1 was defective in the ΔSLC39A9 cells. The reduction in SubAB binding to the cell surface following treatment with swainsonine, a Golgi mannosidase II inhibitor, supports the conclusion that the N-glycan changes in ΔSLC39A9 cells reduce SubAB binding to the cell surface.

Not only complex-type N-glycans but also core 1 O-glycans were markedly reduced in ΔSLC39A9 cells, due to a significant reduction in the corresponding enzyme, C1GalT1 (Figures 5, 6B, and S3F). The ability of SubAB to bind to p135 almost disappeared in ΔSLC39A9 cells, as in ΔC1GalT1 cells (Figures 2D and 3D), which is consistent with the defective O-glycans in ΔSLC39A9 cells. On the other hand, the binding of SubAB to p175 was not affected as much in ΔSLC39A9 cells as in ΔMGAT1 cells, although the binding of SubAB to the cell surface was reduced (Figures 3C and 3D). Together with the result that resistance to SubAB in ΔSLC39A9 cells is slightly greater than that in ΔC1GalT1 cells (Figures 2A and 3A), the reduced sensitivity to SubAB in ΔSLC39A9 cells seems to be due to O-glycan defects in addition to N-glycan defects. The importance of O-glycan defects in ΔSLC39A9 cells in relation to SubAB cytotoxicity is supported by the previous finding that SLC39A9 was not enriched in a ricin screen, in which O-glycan-related genes were not enriched (Tian et al., 2018).

How does SLC39A9 regulate N- and O-glycan biosynthesis? ZIP family proteins are believed to transport zinc as well as iron, manganese, and/or cadmium ions into the cytosol, either across the plasma membrane or from intracellular organelles (Guerinot, 2000, Kambe et al., 2017). We demonstrated that mutation of His-155, predicted to be critical for zinc transport, led to a loss of glycan-regulating activity, supporting the idea that zinc transport by SLC39A9 is involved in glycan regulation. Interestingly, the MAN2A1 and C1GalT1 proteins are known to be associated with Zn^2+^. The MAN2A1 protein has a Zn^2+^-binding site on the Golgi lumen side, and this binding is essential for its activity (Shah et al., 2008, van den Elsen et al., 2001). On the other hand, a direct requirement by C1GalT1 for Zn^2+^ activity has not yet been reported; its chaperon, C1GalT1C1, however, binds to Zn^2+^ at the C-terminal domain that is also required for its oligomerization (Hanes et al., 2017). Therefore zinc homeostasis, controlled by SLC39A9, may also be involved in the activities of MAN2A1 and C1GalT1C1 to regulate both N- and O-glycan biosyntheses. However, this hypothesis contains an apparent contradiction. Most Zip family proteins are generally believed to transport Zn^2+^ either from extracellular fluids or from the lumen of intracellular organelles to the cytoplasm (Eide, 2006, Taniguchi et al., 2013). If the direction of Zn^2+^ transport by SLC39A9 is true, loss of SLC39A9 should mean Zn^2+^ accumulating in the Golgi lumen. However, the Zn^2+^-binding sites of MAN2A1 and C1GalT1C1 face the lumen of the Golgi; therefore it is unlikely that Zn^2+^ deficiency in the Golgi lumen inactivates MAN2A1 and C1GalT1C1 proteins in *SLC39A9* KO cells. Elucidation of the molecular mechanism of SLC39A9, including the direction of movement of zinc or other divalent cation transport, is required to understand the involvement of these divalent cations in glycosylation, which is a subject for future investigations.

Most membrane trafficking genes enriched in the SubAB screen, including the COG complex, the GARP complex, *UNC50*, *GOSR1*, and *PTAR1*, were also observed in an STx screen (Yamaji et al., 2019), a cholera toxin screen (Gilbert et al., 2014), ricin screens (Tian et al., 2018, Bassik et al., 2013), and other screens related to glycoproteins and proteoglycans (Jae et al., 2013, Jae et al., 2014, Riblett et al., 2015, Tanaka et al., 2017). These factors are thought to affect glycosylation, likely through retrograde trafficking defects of glycosyltransferases, or by being required for retrograde trafficking of toxins. In fact, the COG complex affects both glycosylation and toxin transport of STx and SubAB (Zolov and Lupashin, 2005, Zeevaert et al., 2008, Smith et al., 2009). The *JTB* gene was enriched in both SubAB and ricin screens (Figure S1). KO of *JTB* did not affect glycosylation or SubAB binding to surface glycoproteins, but did suppress Bip cleavage (Figures 3B and 3C), suggesting that JTB affects the transport of toxin-binding glycoproteins into the ER. On the other hand, the *KDELR1* and *KDELR2* genes were specifically enriched in the SubAB screen. As with *JTB*, KO of *KDELR2* did not affect glycosylation but did suppress Bip cleavage. Bip has a retrieval motif that is recognized by KDEL receptors (Lewis and Pelham, 1992), and KO of the *KDELR2* gene suppressed Bip cleavage induced by SubAB (Figure 3B). Future research should aim to elucidate whether the interaction of KDELRs with Bip is involved in SubAB-induced cell death.

In summary, we performed a genome-wide loss-of-function screen using a CRISPR library to identify genes that conferred resistance to SubAB and demonstrated that not only N-glycans but also O-glycans serve as receptors for SubAB. Furthermore, SLC39A9, a predicted zinc transporter, regulates both N- and O-glycan biosynthesis at the Golgi complex. These results provide important insights into critical host factors, including receptors for SubAB and post-translational regulation of glycan synthesis.

### Limitations of the Study

As shown in Figure S2, the expression level of O-glycans was as high as N-glycans in HeLa cells. However, the loss of O-glycans did not affect the binding of SubAB to the cell surface according to the FACS analysis (Figure 2C). There are several possible explanations for this. First, N-glycans may have a more preferential glycan structure for SubAB binding compared with O-glycans. Second, it may be difficult for SubAB to access O-glycan-containing proteins depending on the position of the attached sugar chains. Third, if HeLa cells abundantly express mucins that contain many O-glycans, SubAB may be able to bind only to parts of the O-glycans because of limited space, and as a result, the amount of SubAB binding to O-glycans may not be equivalent to the amount of O-glycans. The cell-surface biotin labeling experiment demonstrated that SubAB bound to some proteins in an O-glycan-dependent manner; we therefore think that part of a SubAB molecule binds to an O-glycan on the cell surface. In the future it should be possible to demonstrate the direct binding of SubAB to O-glycosylated proteins on the cell surface.

## Methods

All methods can be found in the accompanying Transparent Methods supplemental file.

## Data and Software Availability

The sgRNA data reported in this study have been deposited to the NCBI GEO and are available under accession number GEO: GSE125650.

## Supplemental Information

Document S1. Transparent Methods and Figures S1–S5

Data S1. Read Count, Normalization, and Statistical Analysis of sgRNA in GeCKO Version 2 Library, Related to Figure 1

Data S2. Summary of Enriched Genes in SubAB-Resistant Cells, Related to Figure 1

Data S3. Gene List of Library Set A/B Statistically Significant in SubAB-Resistant Cells, Related to Figure 1

Data S4. Glycan Analysis of ΔMGAT1, ΔC1GalT1, and Double KO Cells, Related to Figure 2

Data S5. Glycan Analysis of ΔKDELR2, ΔJTB, ΔSLC39A9, and Their Revertant Cells, Related to Figures 4 and 5
