# Supplementary material for: A CRISPR Screen Using Subtilase Cytotoxin Identifies SLC39A9 as a Glycan-Regulating Factor
Source: iScience. 2019 May 8;15:407–20. doi: 10.1016/j.isci.2019.05.005 (PMC6526310; doi:10.1016/j.isci.2019.05.005)
Supplement: Document S1. Transparent Methods and Figures S1–S5 [file mmc1.pdf]

ISCI, Volume *15*

Supplemental Information

A CRISPR Screen Using Subtilase Cytotoxin Identifies SLC39A9 as a Glycan-Regulating Factor

Toshiyuki Yamaji, Hisatoshi Hanamatsu, Tsuyoshi Sekizuka, Makoto Kuroda, Norimasa Iwasaki, Makoto Ohnishi, Jun-ichi Furukawa, Kinnosuke Yahiro, and Kentaro Hanada

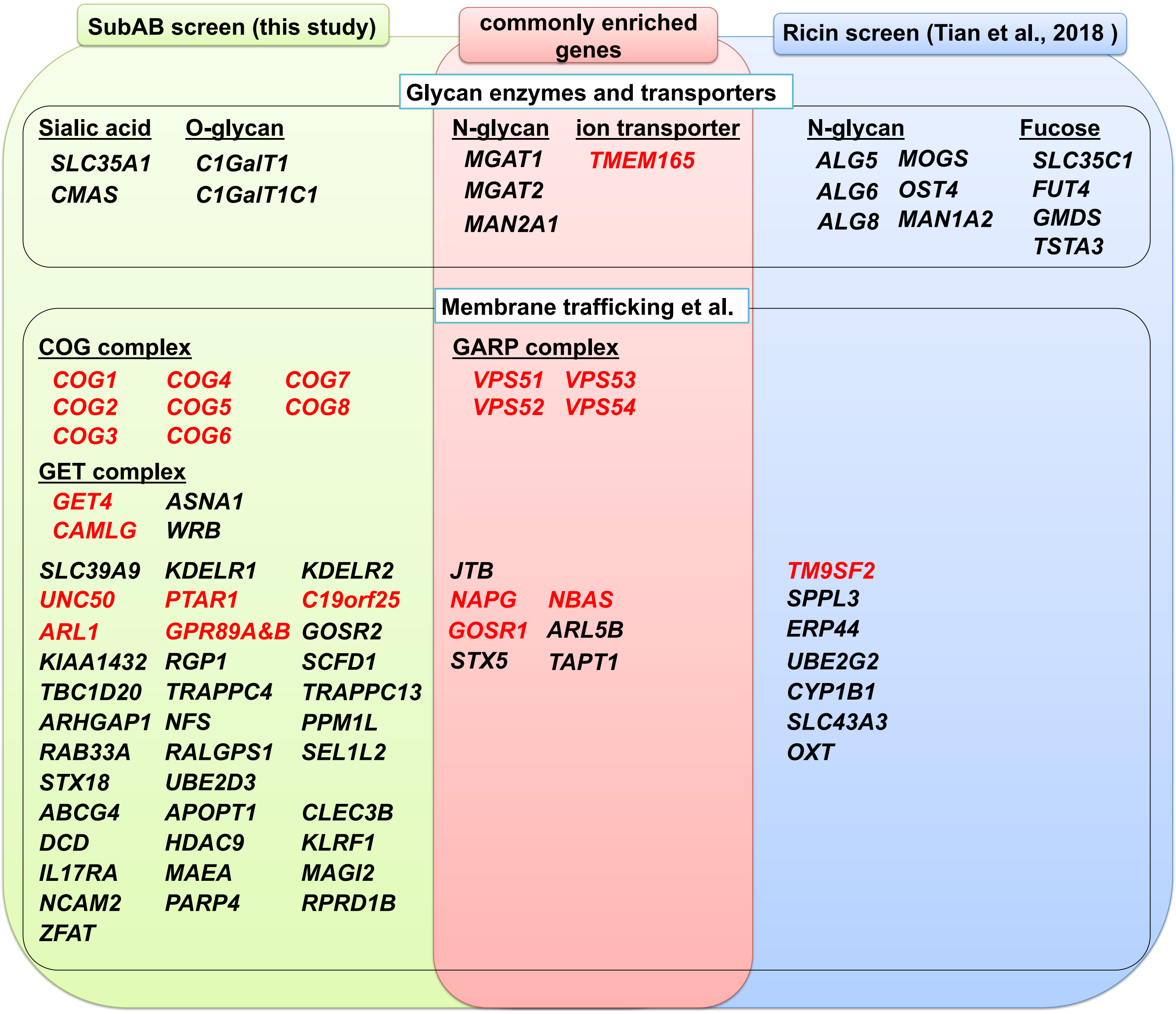

Figure S1. Comparison of Enriched Genes in the SubAB Screen with those in a Ricin Screen, Related to Figure 1. Enriched genes in the SubAB screen were compared with those in a previously reported ricin screen (Tian et al., 2018). Common genes enriched in the two screens are shown in the middle. Genes in red letters are indicative of those enriched in the STx screen (Yamaji et al., 2019). Note that the criteria for enrichment of genes were different between these two screens.

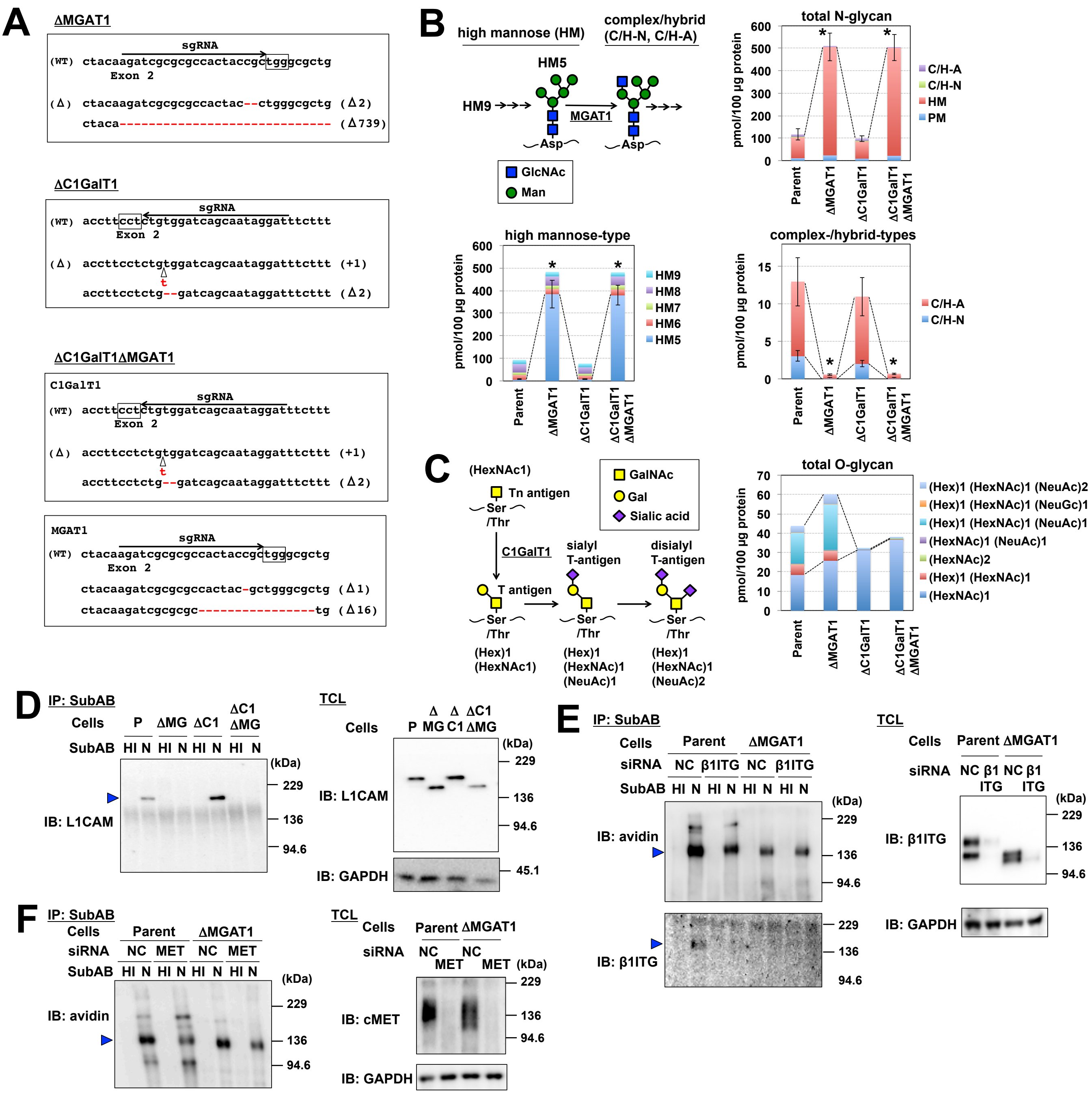

Figure S2. N- and O-glycans Serve as Receptors for SubAB, Related to Figures 1 and 2.

(A) Construction of *MGAT1*-, *C1GalT1*-, and both KO HeLa cells. Red letters in sequences indicate deletion or insertion mutations, which cause frameshifts shown at the right side of the sequences. Boxes are indicative of protospacer adjacent motif (PAM) sequences. (B) N-glycan analysis in KO cells shown in (A). Expression levels of N-glycan species were compared among the indicated cells. Data are the mean values ± SD obtained from three independent experiments. C/H-A indicates acidic complex-/hybrid-type glycans. C/H-N indicates neutral complex-/hybrid-type glycans. HM indicates high mannose-type glycans. PM indicates pauti mannose-type glycans. The biosynthetic step by MGAT1 is shown at the upper left. The Bonferroni-corrected *t*-test was used for multiple comparisons of HM (in the “total N-glycan” graph), HM5 (in the “high mannose-type” graph), and C/H-A and C/H-N (in the "complex-/hybrid-types” graph). * p <0.017. (C) O-glycan analysis in KO cells shown in (A). Expression levels of O-glycan species were compared among the indicated cells. Data are the mean values obtained from three independent experiments. Core 1 O-glycan biosynthesis is shown at the left. (D-F) Binding of SubAB to L1CAM (D), β1 integrin (β1ITG) (E), and MET (F) in glycan KO cells. Biotinylated cell-surface proteins prepared from the indicated cells were immunoprecipitated with heat-inactivated (HI) or wild-type SubAB (N) as described in the Supplemental Experimental Procedures section. SubAB-binding proteins were detected with streptavidin-HRP or the indicated antibodies. TCL indicates total cell lysates. In (E) and (F), knockdown of β1ITG (E) and MET (F) by RNAi was performed. NC indicates the negative control. Triangles indicate the indicated proteins.

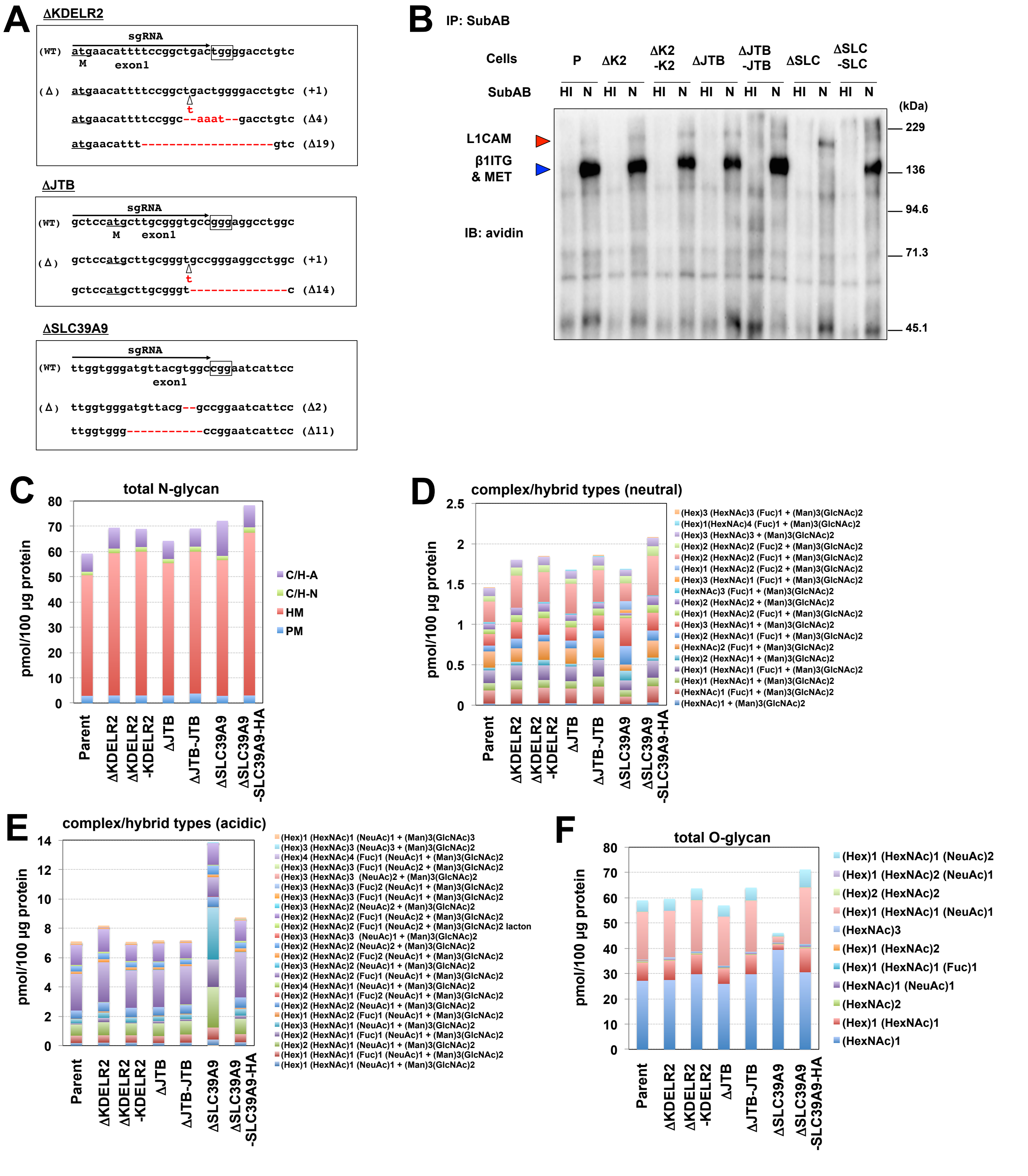

Figure S3. SLC39A9 is Required for N- and O-glycans, Related to Figures 3–5.

(A) Construction of *KDELR2*-, *JTB*-, and *SLC39A9*-KO HeLa cells. Red letters in sequences indicate deletion or insertion mutations, which cause frameshifts shown at the right side of the sequences. Boxes are indicative of protospacer adjacent motif (PAM) sequences. (B) Detection of SubAB-binding proteins. Biotinylated cell-surface proteins prepared from the indicated cells were immunoprecipitated with heat-inactivated (HI) or wild-type SubAB (N) as described in the Supplemental Experimental Procedures section. SubAB-binding proteins were detected with streptavidin-HRP. (C–F) Quantitative glycan analysis. Expression levels of total N-glycans (C), neutral complex-/hybrid-type N-glycans (D), acidic complex-/hybrid-type N-glycans (E), and total O-glycans (F) in the indicated cells were plotted on graphs. The full raw data set is shown in Data S5 and expression levels of respective glycan species were plotted on graphs in Figures 4 and 5. Data are the mean values obtained from eight independent experiments.

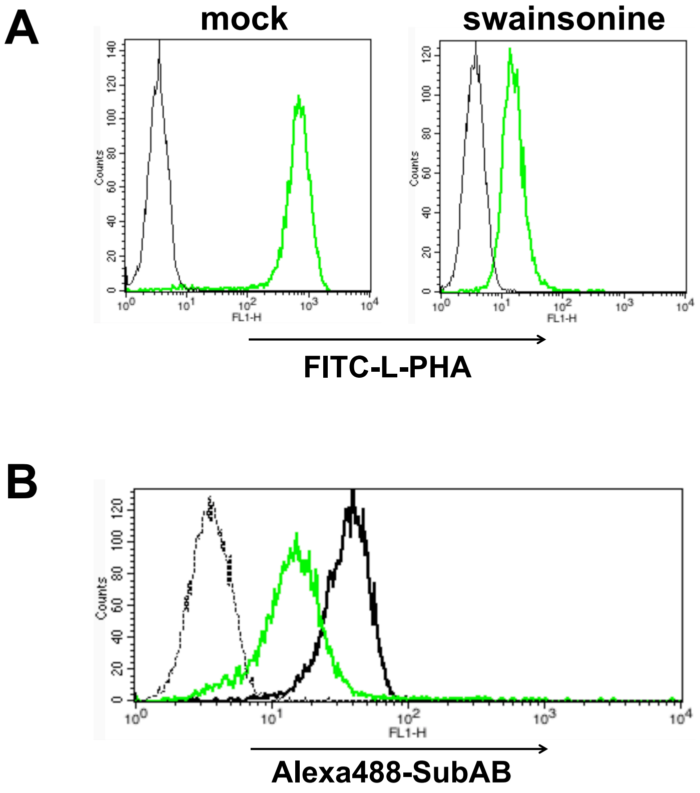

Figure S4. Effect of a Golgi Mannosidase Inhibitor on the Surface Binding of SubAB, Related to Figure 4.
HeLa cells were treated with 1 µg/ml swainsonine or mock solution (DMSO) for 3 days. (A) Cells were stained with (yellow–green lines) or without (black lines) FITC-labeled L-PHA, which recognizes β1,6GlcNAc-branching N-glycans, and analyzed using FACS. (B) Mock-treated cells (black lines) and swainsonine-treated cells (yellow–green line) were stained with (bold lines) or without (dotted line) Alexa488-labeled SubAB, and analyzed using FACS.

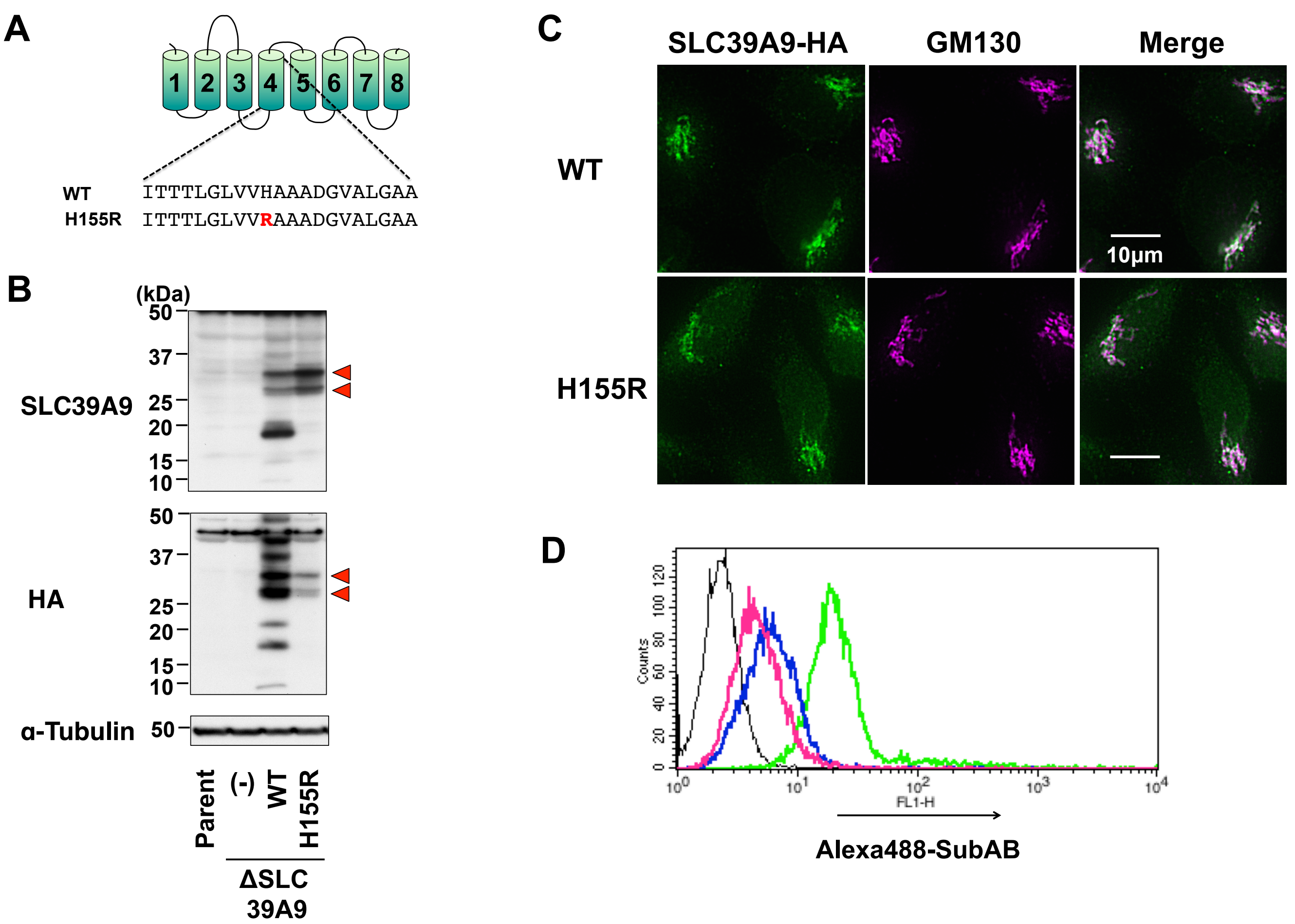

Figure S5. The 155th Histidine in SLC39A9 is Required for the Binding of SubAB to Cell Surfaces, Related to Figure 6.

(A) Replacement of His155 with Arg (H155R) in the 4th transmembrane domain in SLC39A9. (B) Expression of C-terminal HA-tagged wild-type and H155R mutated SLC39A9 in *SLC39A9* KO cells. The proteins were detected by immunoblots using anti-SLC39A9 and anti-HA. Arrowheads are indicative of SLC39A9 proteins with the expected size. (C) Intracellular localization of wild-type SLC39A9-HA and SLC39A9 H155R-HA. ΔSLC39A9/SLC39A9-HA and ΔSLC39A9/SLC39A9 H155R-HA cells were stained with anti-HA antibodies and anti-GM130 (Golgi marker). Scale bars = 10 µm. (D) Surface binding of SubAB. Parent cells (yellow-green line), ΔSLC39A9 cells (blue line), ΔSLC39A9/SLC39A9 H155R-HA cells (magenta line) were stained with Alexa488-labeled SubAB (Alexa488-SubAB) and analyzed using FACS. The black line indicates no staining in ΔSLC39A9/SLC39A9 H155R-HA cells.

TRANSPARENT METHODS

Cell Culture, Antibodies, and Reagents

The HeLa-mCAT#8 clone, which expresses mouse cationic amino acid transporter 1 (which serves as the mouse ecotropic retroviral receptor) (Yamaji et al., 2010), and its KO mutants and transfectants were maintained in Dulbecco's modified Eagle's medium (DMEM) containing 10% heat-inactivated fetal bovine serum (FBS) and 4.5 g/L glucose at 37 °C in a 5% CO_2 incubator. 293FT cells (Thermo Fisher, Rockford, USA) used for lentivirus production were maintained in DMEM containing 10% FBS with non-essential amino acids and sodium pyruvate. Plat-E cells (Morita et al., 2000) for retrovirus production were maintained in DMEM containing 10% FBS with 1 μg/ml puromycin and 10 μg/ml blasticidin-S.

Purchased antibodies (Abs) were as follows: rabbit anti-SLC39A9 Abs (#ab137205) and mouse anti-alpha Tubulin IgG (#ab7291) (Abcam, Cambridge, UK), rat anti-HA IgG (#H6533) and horseradish peroxidase (HRP)-rat anti-hemagglutinin (HA) tag IgG (Sigma-Aldrich, St. Louis, MO), mouse anti-GM130 IgG (#610823) and mouse anti-BiP IgG (#610978) (BD Transduction Laboratories, San Diego, USA), rabbit anti-GAPDH Abs (GTX100118) (GeneTex, Irvine, USA), rabbit anti-L1CAM Abs (#eBio5G3) (Thermo Fisher), mouse anti-β1 integrin Abs (P5D2) (Santa Cruz BioTechnology, Dallas, USA), mouse anti-MET Abs (#3127) (Cell Signaling, Danvers, USA). Streptavidin-HRP (RPN1231) was purchased from GE Healthcare (Buckinghamshire, UK). FITC-conjugated L-PHA was purchased from J-oil mills (Tokyo, Japan). Alexa-conjugated secondary antibodies were purchased from Thermo Fisher.

3-(4,5-Dimethylthiazoyl-2-yl)-2,5-diphenyltetrazolium bromide (MTT) and puromycin were purchased from Sigma. Blasticidin-S was purchased from Kaken Pharmaceutical (Tokyo, Japan). Lipofectamine LTX reagent was purchased from Thermo Fisher. Polyethylenimine Max (PEI-Max) was purchased from Polysciences Inc (Warrington, USA). Swainsonine was purchased from Wako (Tokyo, Japan). The human Genome-scale CRISPR Knock-Out (GeCKO) v2.0 library in the lentiGuide-Puro plasmid (65,386 single-guide RNAs (sgRNAs) in library A and 58,031 sgRNAs in library B) and the lentiCas9-Blast plasmid (two vector lentiviral GeCKO system) were obtained from Addgene (Sanjana et al., 2014). Primers used in this study are described at the end of the "Methods".

Preparation of SubAB and Fluorescence-labeled SubAB

Recombinant His-tagged SubAB and catalytically inactive mutant $SubA_{S272A}B$ were synthesized in *E. coli* BL21

(DE3) and purified under native conditions using Ni-nitrilotriacetic acid (NTA) agarose (QIAGEN, Venlo, Netherlands) chromatography (Morinaga et al., 2007). Purified SubAB was labeled with Alexa488 (Thermo Fisher) according to the manufacturer's instruction manual.

CRISPR Screen for SubAB Treatment

Four sgRNA-expressing HeLa cell libraries (A-1, A-2, B-1, and B-2) using GeCKO v2.0 library (Libraries A and B) were prepared and stored (Yamaji et al., 2019). 3×10^7 sgRNA-expressing cells from each cell library were plated 24 h prior to treatment with SubAB. Then, cells were washed with DMEM (containing no FBS) and treated with 7.5 ng/ml SubAB in DMEM for 24 h. The next day, cells were washed with DMEM containing 10% FBS and cultured in conventional conditions for 11 days. Surviving cells were then re-plated and treated with 10 ng/ml SubAB again in DMEM for 24 h. Five days after treatment, cells were trypsinized and frozen as cell pellets. For untreated controls, 1.2×10^7 sgRNA-expressing cells in each cell library were cultured for the same period as SubAB-treated cells with several passages, such that a minimum of 1.2×10^7 cells was present in each passage.

Genomic DNA Sequencing

Analysis of genome-integrated sgRNAs was performed as follows (Yamaji et al., 2019): genomic DNA from frozen cells was purified using the conventional phenol-chloroform method. Amplification of the genome-integrated sgRNA sequences by PCR was performed as follows, based on a previous method (Shalem et al., 2014). For the first PCR, 100 µg genomic DNA from untreated cells or SubAB-treated cells was used as a PCR template. For each sample (A-1, A-2, B-1, and B-2), nine separate 100 µl reactions were performed using PrimeStar GXL DNA polymerase (Takara, Otsu, Japan) and the following primers (9 forward primers and 1 reverse primer):

(Fw) 1stY1R1s0–8: CTACACGACGCTCTTCCGATCT (0–8 bp random sequence for increasing library complexity) TCTTGTGGAAAGGACGAAACACCG

(Rv) 1stY2as: GCCACTTTTTCAAGTTGATAACGGACTAG

Amplification was carried out over 20 cycles; 1 µl from each of the nine separate first PCR products was used as a template for the second round of PCR. For each sample, nine separate 20 µl PCR reactions were performed using the following primers:

(Fw) 2nd P5R1s: <u>AATGATACGGCGACCACCGAGATCTACA</u>**CTCTTTCCCTACACGACGCTC TTCCGATCT**

(Rv) 2nd P7Y2as: <u>CAAGCAGAAGACGGCATACGAGAT</u> (CC (A-1), or TT (A-2), or AA (B-1), or GG (B-2) as

barcodes for multiplexing of different samples) GCCACTTTTTCAAGTTGATAACGGACTAG

Illumina adaptor sequences (P5 and P7 respectively) are underlined, while the sequence primer site for MiSeq sequence analysis is indicated by bold font. Amplification was carried out over 10 cycles. The resulting nine amplicons in each sample were mixed and gel was extracted using SYBR Gold (Thermo Fisher). The extracted DNA was then quantified using a Quantus fluorometer (Promega, Madison, USA), as well as running an agarose gel with 100 bp quantifiable DNA Ladder (NEB, Ipswich, USA), and equal amounts of each sample (A-1, A-2, B-1, and B-2) were mixed. The DNA concentration of the mixture was adjusted for sequencing analysis. PhiX Control Kit v3 (Illumina San Diego, USA) was added to the sample at approximately 20% concentration. MiSeq Reagent Kit v3 (Illumina) was used for MiSeq sequencing (Illumina).

Data Processing and Analysis

Data processing and analysis was performed as follows (Yamaji et al., 2019): to perform demultiplexing of fastq sequence data, total raw read sequences were divided into each sample with barcode sequences of "AA", "CC", "GG", and "TT", using an in-house program. The adapter sequences were removed using a skewer program (version 0.1.126) (Jiang et al., 2014) with the following parameters: minimum read length = 10 mer, maximum read length = 30 mer, lowest mean quality value = 19 sanger quality score. To extract high-quality sgRNA sequences, sequences with a Phread quality score less than 20 were excluded using the "split_libraries_fastq.py" (version 1.9.1) function of the QIIME program (Caporaso et al., 2010). The number of sgRNA sequences was calculated with "sort" and "uniq" of the unix command program, followed by normalization with the following formula; normalized reads per sgRNA = reads per sgRNA / total reads for all sgRNAs in sample × 10^7 (Data S1). Fold enrichment was calculated using the following formula: Fold enrichment = normalized reads in SubAB-treated sample / normalized reads in untreated sample. When the normalized reads in the untreated sample equaled 0, fold enrichment was calculated by setting 0 to 1. First, essential genes closely related to SubAB interactions were identified using the MAGeCK program (version 0.5.7) (Li et al., 2014) to analyze normalized sgRNA count data (Data S3). Using this program, 735 genes contained at least one significantly different sgRNA. For stricter selection of hit sgRNAs, the sgRNAs representing more than 1-fold enrichment in both independent cell libraries (A-1 and A-2, or B-1 and B-2) were selected as SubAB resistance sgRNA candidates (Data S2), and fold enrichment of these candidates was plotted on graphs (Figure 1A). Note that the selected sgRNAs were all statistically significantly enriched, which was demonstrated using the MAGeCK program (Data S1 and S2).

Synthesis of CRISPR Plasmids and Construction of CRISPR KO Cell Lines

The pSELECT-CRISPR-Cas9 plasmid (Ogawa et al., 2018, Yamaji et. al. 2019) was cleaved with BsmBI, and a 20 mer guide sequence was ligated into the site. The sgRNA sequences used in this study are described at the end of the Method section. A CRISPR plasmid was mixed with X-tremeGENE HP (Roche Diagnostics) (1 μg of plasmid and 2 μl X-tremeGENE HP were mixed in 100 μl Opti-MEM), and the mixture was then added to HeLa-mCAT8 cells (1.5 × 10^5 cells/well in 12-well plates one day before transfection). The next day, cells were transferred to 6-well plates and cultured at 37°C with puromycin at 5 μg/ml for 3 days to exclude the untransfected cells. After 3 days, the culture medium was changed to puromycin-free medium, and the cells were subcultured for 3 days. CRISPR-treated HeLa cells were used for SubAB treatment, and the cell viability assay was conducted as described below (Figure 1B), harvested for indel analysis, or diluted to isolate gene-disrupted clones.

Indel Analysis

Indel analysis was performed as follows (Yamaji and Hanada, 2014): trypsinized cells were heated in TE buffer followed by vortexing, to use as a template for genomic PCR. PCR was performed with PrimeSTAR GXL, and blunt-end PCR products were directly sequenced or cloned with a Zero Blunt TOPO PCR Cloning Kit (Invitrogen) to use as a template for sequence analysis. Clones in which all alleles of the target gene had frameshift-causing mutations were selected as KO cells. In this study, KO cell clones of *MGAT1*, *C1GalT1*, both *C1GalT1* and *MGAT1*, *KDELR2*, *JTB*, and *SLC39A9* were isolated.

SubAB Treatment and Cell Viability Assay

For SubAB treatment, cells (5–10 × 10^3 cells/ml in 12- or 24-well plates) were cultured overnight at 37°C, and then incubated for 24 h with the indicated concentrations of SubAB in DMEM (no FBS). After washing, the cells were cultured in DMEM with 10% FBS for an additional four days. To assess cell viability, an MTT assay was performed as follows (Yamaji et al., 2010): cells were incubated with 0.25 mg/ml MTT in the culture medium for 2 h at 37°C in a CO_2 incubator. After removal of the MTT-containing medium, cells were lysed with 200–300 μl of 0.4 N HCl in isopropanol. The absorbance at 570 nm was measured using an Opsis microplate reader (Dynex Technologies, Chantilly, USA). Cell viability was expressed as a percentage of the value (OD570) in the absence of SubAB.

RNA isolation, Reverse Transcription (RT) PCR, and Real-time PCR

Total RNA was isolated using TRIzol Reagent as per the manufacturer's instructions (Thermo Fisher). RT-PCR was

performed using ReverTra Ace qPCR RT Master Mix with gDNA Remover (ReverTra Ace, Toyobo, Japan) and 0.75 μg total RNA in 15 μl reaction mixture. For real-time PCR, a LightCycler 96 system with LightCycler-FastStart DNA master SYBR Green I kit (Roche) was used according to the manufacturer's protocol (Yamaji et al., 2010). GAPDH mRNA was used as an internal control. After normalizing with GAPDH, relative mRNA levels of C1GalT1 and MAN2A1 were expressed as a percentage of the value in parent cells.

Retroviral Infection and Preparation of Stable Transfectants

Human *KDELR2*, *JTB*, and *SLC39A9* cDNAs were amplified by PCR (template: HeLa mCAT#8 cDNA), then amplified DNAs were digested with restriction enzymes and inserted into a retroviral plasmid, pMXs-IB. An HA-tag sequence was attached just before the STOP codon of *SLC39A9* cDNA. Preparation of retroviruses and infection of HeLa-mCAT#8-based cells was performed using the Plat-E system as follows (Morita et al., 2000, Yamaji et al., 2010): plasmids were transfected into Plat-E cells using Fugene 6 (Promega, Fitchburg, USA). The next day, the culture medium was changed and the cells were cultured for a further 24 h. The medium containing retroviruses was filtered using a 0.45 μm filter (Millipore) and stored at -80°C until use. The viral supernatant, mixed with 5 μg/ml polybrene, was added to the HeLa-mCAT#8-based cells and incubated at 37°C for 4 h. After viruses were removed, the cells were cultured for a further 24–48 h. Then, blasticidin-S (7.5 μg/ml) was added for selection. Established cells were as follows: ΔKDELR2/KDELR2, ΔJTB/JTB, and ΔSLC39A9/SLC39A9-HA.

Immunofluorescence Microscopy

Immunostaining was performed as follows (Kawano et al., 2006): cells were grown on a glass coverslip in a 6-well plate for 72 h. The cells were fixed with Mildform 10N (Wako) for 20 min at room temperature. After washing twice with PBS, the cells were sequentially incubated at room temperature with 0.1 M NH_4Cl in PBS for 20 min and with 0.1% Triton X-100 for 15 min. After washing twice with PBS, the cells were incubated with 3% bovine serum albumin (BSA) in PBS for 30 min. The cells were then incubated with rat anti-HA IgG and mouse anti-GM130 IgG for 1 h. After washing three times with PBS, the cells were incubated with Alexa488-conjugated anti-rat IgG and Alexa594-conjugated anti-rat IgG for 1 h. After washing three times with PBS, the coverslips were mounted on Fluoromount (Diagnostic Biosystems, Pleasanton, USA). The specimens were visualized using a wide-field fluorescence microscope, BZ-X700 (Keyence, Osaka, Japan), equipped with a Plan Apo VC 60x1.20 WI (water immersion) objective. A haze reduction function (condition 2), which applies a no-neighbor deconvolution algorithm to the captured image, was used to eliminate fluorescence blurring caused by scattered light and capture clear images

with high contrast.

Western Blot Analysis

For SLC39A9 detection, cells were scraped and sonicated in sonication buffer (10 mM Hepes/NaOH (pH 7.4) 1 mM EDTA, 0.25 M sucrose, and protease inhibitor cocktail) and subsequently mixed with Laemmli sodium dodecyl sulfate (SDS) sample buffer. Protein concentrations were determined using the Pierce BCA protein assay kit using BSA as a standard. Proteins were resolved by SDS-PAGE, transferred to Immobilon-P PVDF membranes (Millipore, Darmstadt, Germany) using the wet transfer method, and probed with specified antibodies. Antigen signals were detected using SuperSignal West Femto Maximum Sensitivity Substrate (Thermo Fisher) or Chemi-Lumi One L (Nacalai, Kyoto, Japan) and exposed to an X-ray film.

Detection of BiP Cleavage Induced by SubAB

Cells (2–3 × 10^5/well) in a 48-well plate (Thermo Fisher) were cultured overnight in 300 μl of EMEM containing 10% FBS. Cells were incubated with the indicated concentration of wild-type SubAB (wt) or catalytically inactive mutant SubAB (mt) for 12 h at 37°C. Cells were mixed with 100 μl of SDS-sample buffer, and heated to 100°C for 10 min. Following SDS-PAGE, proteins were transferred to PVDF membranes, and probed with anti-BiP monoclonal antibodies or anti-GAPDH antibodies. Antigen signals were detected using EzWestLumiOne (ATTO, Tokyo, Japan) and visualized by LAS-1000 (Fuji Film).

Immunoprecipitation Analysis

Immunoprecipitation of SubAB binding proteins from HeLa cell surfaces was performed as follows (Yahiro et al., 2011): cells were harvested using TNE buffer (40 mM Trsi-HCl, pH 7.5, 0.15 M NaCl, and 1 mM EDTA) and then washed twice with cold PBS. Cell surface proteins were biotinylated with biotinamidohexanoic acid N-hydroxysuccinimide ester (Sigma). The biotinylated cells were lysed with Sol buffer (50 mM Tris-HCl (pH 7.5), 100 mM NaCl, 10% glycerol, 1% Triton X-100, and protease inhibitor cocktail [Roche Diagnostics, Mannheim, Germany]). The lysates (100 μg/200 μl) were incubated at 4°C for 1 h with 1 μg native SubAB (N) or heat-inactivated SubAB (HI), followed by incubation at 4°C overnight with 1 μl rabbit anti-SubAB antibodies (Yahiro et al., 2006). The immune complexes were collected after being incubated at 4°C for 1.5 h with 20 μl Protein G agarose (Thermo Fisher) 50% (v/v) in Sol buffer. Afterwards the beads were washed three times with Sol buffer.

The bound proteins were solubilized using 1× SDS-PAGE sample buffer. Following SDS-PAGE and transfer to a PVDF membrane, proteins were probed with HRP-streptavidin or the indicated antibodies.

FACS Analysis

Non-confluent cells were dissociated using Cell Dissociation Buffer (Thermo Fisher). Cells were washed with wash buffer (1% BSA in PBS) and incubated with Alexa488-SubAB or FITC-L-PHA in wash buffer for 45 min on ice. After washing with wash buffer once, cells were analyzed using a FACSCalibur analyzer (BD Biosciences, Franklin Lakes, USA).

N-Glycan Preparation

Approximately 4×10^6 cells were cultured overnight at 37°C. The next day, cells were washed 5 times with cold PBS, and collected using a scraper. The pelleted cells were frozen until analysis. Pelleted cells were suspended in 100 μL of H_2O and homogenized using an Ultrasonic Homogenizer (TAITEC, Saitama, Japan). Ethanol (400 μL) was added to homogenized cells which were then placed in a freezer at -30°C for 16 h. The cellular pellet and supernatant fractions were separated by centrifugation, then the cellular pellet was dissolved in 100 μL of water and the protein concentration was measured using a BCA protein assay kit (Thermo Fisher). The pellet fraction containing 50 μg proteins was treated with tris(2-carboxyethyl)phosphine (Sigma) and 2-iodoacetamide (Sigma) in 100 mM ammonium bicarbonate containing 0.1% triton-X (Sigma). After reductive alkylation, proteins were digested with trypsin (Sigma) at 37°C for 16 h. The reaction mixture was heated to 90°C for 10 min to inactivate tre trypsin. Deglycosylation was carried out by the addition of 2 U of PNGase F (Sigma).

Glycoblotting of N-Glycans

Cellular N-glycan analysis was subjected to a glycoblotting procedure as previously described (Fujitani et al., 2011). In brief, the PNGase digested sample (25 μg protein) containing internal standards of Neu5Ac2Gal2GlcNAc2 + Man3GlcNAc1 (A2GN1, 10 pmol) was captured on 5 mg BlotGlyco® beads (Sumitomo Bakelite Company Ltd. Tokyo, Japan). Unreacted hydrazide groups on beads were capped by acetylation with 10% acetic anhydride in methanol. To modify carboxy groups of sialylated glycan, methyl esterification was performed with 100 mM 3-methyl-1-p-tolyltriazene (TCI, Tokyo, Japan) in dioxane. Next, these methyl-esterified glycans were released and labeled with aoWR via transimination. Excess aoWR reagent was removed using a HILIC μElution plate (Waters, Milford, USA). Finally, the purified N-glycans were eluted with 1% acetic acid and 5% acetonitrile in water,

followed by MALDI-TOF MS analysis.

O-Glycan Preparation

Proteins equivalent to 100 μg were purified using an Amicon Ultra Centrifugal Filter unit (molecular-weight cutoff, 3 kDa) (Millipore, Burlington, USA). The purified proteins were subjected to a microwave-assisted β-elimination in the presence of pyrazolone analogues (BEP) reaction at 120°C for 2 h using a Monowave 300 microwave reactor (Anton Paar Japan, Tokyo, Japan). After the reaction, 20 pmol bis-PMP-labeled N, N′, N″, N‴-tetraacetyl chitotetraose (GN4) was added as an external standard, and the mixture was neutralized with 1.0 M HCl. Chloroform was added to the reaction mixture which was then vigorously stirred, and the aqueous layer was recovered for removal of excess reagent. The aqueous layer containing PMP-labeled glycans was purified by passage through a graphitized carbon column and an Iatrobeads silica gel column, as previously described (Furukawa et al., 2015a).

Tandem MALDI-TOF MS Analysis

MALDI-TOF MS analysis was performed as previously described (Furukawa et al., 2015b). Briefly, all measurements were performed using an Ultraflex II TOF/TOF mass spectrometer equipped with a reflector and controlled by the FlexControl 3.0 software package (Bruker Daltonics, Bremen, Germany), according to general protocols. All spectra were obtained in the reflectron mode with an acceleration voltage of 25 kV, a reflector voltage of 26.3 kV, and a pulsed ion extraction of 160 ns in positive-ion mode. Absolute quantification was performed by comparative analyses between the areas of the MS signals derived from each N- and O-glycan and a known amount of the internal standards (A2GN1 and GN4).

Statistical Analysis

For multiple comparisons, the Student's *t*-test with Bonferroni was used. A *p*-value of < 0.017 (0.05 divided by 3) was considered to be statistically significant in three comparisons (Figures 2B and S2B [*MGAT1*, *C1GalT1*, and *C1GalT1* / *MGAT1* KO cells to the parent cells], Figure 3B [*KDELR2*, *JTB*, and *SLC39A9* KO cells to the parent cells], and Figure 6B [*SLC39A9* KO cells to the parent cells, wild-type SLC39A9-expressing *SLC39A9* KO cells to *SLC39A9* KO cells, and H155R mutant-expressing *SLC39A9* KO cells to wild-type SLC39A9-expressing *SLC39A9* KO cells]). A *p*-value of < 0.0083 (0.05 divided by 6) was considered to be statistically significant in six comparisons (Figure 3A [*KDELR2*, *JTB*, and *SLC39A9* KO cells to the parent and the corresponding revertant cells]). In Figures 4 and 5, when the *p*-value produced by the *t*-test between parent cells (#1) and *SLC39A9* KO cells (#6), or *SLC39A9*

KO cells (#6) and its revertant cells (#7) was < 0.0083 (0.05 divided by 6, which means 6 comparisons including #2 to #1, #4 to #1, #6 to #1, #2 to #3, #4 to #5, #6 to #7), the difference was considered to be statistically significant. When the p-value was < 0.025 (0.05 divided by 2, which means 2 comparisons including #6 to #1 and #6 to #7), the difference was considered to be statistically significant. In Figure 1B, the Holm-Bonferroni sequential correction (Holm, 1979) was used for 11 comparisons (11 genes to the mock), with $p_i < 0.05 / (11\text{-}i+1)$. p_1 (the smallest p) < 0.0045 (0.05 divided by 11) and p_{11} (the largest p) < 0.05 (0.05 divided by 1).

Primers Used in this Study

Primers for indel analysis

hMGAT1s: CATCATCGTTAGCCAGGACTGCG
hMGAT1as: GGATAGGTGGCCCGAAAGTACT
hC1GalT1s: CCAGAATTTTTACTCCGGTTATGTATACAGC
hC1GalT1as: ATCATCTGAATGCCTTGCATGAGGATC
hKDELR2 5UTRs: ATCTCGCCATCTTCGCCGCTTC
hKDELR2 Ex1as: GAGCGCGTCTTCCAGATCTTC
hJTB 5UTRs: TGCAGAGTAAGTGCCGCCTC
hJTB Ex1as: CAGAGCTTTAAGGTGAAAGCACAGAG
hSLC39A9s: GCCACTGGAAATTTGTTGTCTAGTGGTTG
hSLC39A9as: CTAGGGAAGCAGCTTCCCTTTGC

Primers for constructing expression vectors (Underlined sequences are indicative of restriction enzyme cutting sites)

hKDELR2 Bam-ATGs: ACCGGATCCGCCATGAACATTTTCCGGCTG
hKDELR2 Xho-STOPas: ACCCTCGAGTTATGCTGGCAAACTGAGCTTCTTTCC
hJTB Bam-ATGs: ACCGGATCCTCCATGCTTGCGGGTGCC
hJTB Xho-STOPas: ACCCTCGAGCTATATGGACTCGATTTGCTTCCGG
hSLC39A9 Bam-ATGs: ACCGGATCCAGAATGGATGATTTCATCTCCATTAGCCTGC
hSLC39A9 Xho-ENDas: ACCCTCGAGATGCTGGTGTCCTACTGACAGGATG
hSLC39A9 H155Rs: GGGTCTGGTTGTCC**G**TGCTGCAGCTGATGG (bold: mutation site)
hSLC39A9 H155Ras: CCATCAGCTGCAGCA**C**GGACAACCAGACCC (bold: mutation site)

Primers for real-time PCR

C1GalT1 s1: GCAAGGCATTCAGATGATAATGGAC
C1GalT1 as1: CCAAGTAGCTTTGACGTGTTTGG
MAN2A1 s1: GAGACTCAGTCATCAATTTGAGTGAG
MAN2A1 as1: CAAACTCCACCATCTGGATTGTC

sgRNA Target Sequences

Figure 1B and Figures S2 and S3

MGAT1: AGATCGCGCGCCACTACCGC

C1GalT1: ATCCTATTGCTGATCCACAG

KDELR1: CTTCGACCTCATCGCCATTG

KDELR2: AAGCCAGCTTCTGTTTGCAC

JTB: CCAAGCAGAGGCTCCCGTGC

SLC39A9: CTGGCAGTCATCGTGCCTGA

SLC35A1: TATAACTTCTGTGATACACA

UNC50: AATCTATGAGTACAACCCAA

COG4: TCTAGGGATTGCCCGCATTG

VPS54: TACTTGCTCCAGATCTGTCC

GET4: ACGAGGCGCACCAGATGTAC

Plasmids Constructed in this Study

pMXs-IB-KDELR2: cDNA (BamHI-XhoI), plasmid (BamHI-XhoI)

pMXs-IB-JTB: cDNA (BamHI-XhoI), plasmid (BamHI-XhoI)

pMXs-IB-SLC39A9-HA: cDNA (BamHI-XhoI), HA (XhoI-NotI), plasmid (BamHI-NotI)

pMXs-IB-SLC39A9-H155R-HA: cDNA (BamHI-XhoI), HA (XhoI-NotI), plasmid (BamHI-NotI)

SUPPLEMENTAL REFERENCES

Caporaso, J.G., Kuczynski, J., Stombaugh, J., Bittinger, K., Bushman, F.D., Costello, E.K„ Fierer, N., Peña, A.G., Goodrich, J.K., Gordon, J.I., et al. (2010). QIIME allows analysis of high-throughput community sequencing data. Nat. Methods 7, 335-336.

Fujitani, N., Takegawa, Y., Ishibashi, Y., Araki, K., Furukawa, J., Mitsutake, S., Igarashi, Y., Ito, M., and Shinohara, Y. (2011). Qualitative and quantitative cellular glycomics of glycosphingolipids based on rhodococcal endoglycosylceramidase-assisted glycan cleavage, glycoblotting-assisted sample preparation, and matrix-assisted laser desorption ionization tandem time-of-flight mass spectrometry analysis. J. Biol. Chem 286, 41669-41679.

Furukawa, J.-i., Piao, J., Yoshida, Y., Okada, K., Yokota, I., Higashino, K., Sakairi, N., Shinohara, Y. (2015a). Quantitative O-Glycomics by Microwave-Assisted β-Elimination in the Presence of Pyrazolone Analogues. Anal. Chem. 87, 7524-7528.

Furukawa, J., Sakai, S., Yokota, I., Okada, K., Hanamatsu, H., Kobayashi, T., Yoshida, Y., Higashino, K., Tamura, T., Igarashi, Y., and Shinohara, Y. (2015b). Quantitative GSL-glycome analysis of human whole serum based on an EGCase digestion and glycoblotting method. J. Lipid Res. 56, 2399-2407.

Holm, S. (1979). A simple sequentially rejective multiple test procedure. Scand J Statist. 6, 65–70.

Jiang, H., Lei, R., Ding, S.W., and Zhu, S. (2014). Skewer: a fast and accurate adapter trimmer for next-generation sequencing paired-end reads. BMC Bioinformatics 15, 182.

Kawano, M., Kumagai, K., Nishijima, M., and Hanada, K. (2006). Efficient trafficking of ceramide from the endoplasmic reticulum to the Golgi apparatus requires a VAMP-associated protein-interacting FFAT motif of CERT. J. Biol. Chem. 281, 30279-30288.

Li, W., Xu, H., Xiao, T., Cong, L., Love, M.I., Zhang, F., Irizarry, R.A., Liu, J.S., Brown, M., and Liu, X.S. (2014). MAGeCK enables robust identification of essential genes from genome-scale CRISPR/Cas9 knockout screens. Genome Biol. 15, 554.

Morinaga, N., Yahiro, K., Matsuura, G., Watanabe, M., Nomura, F., Moss, J., and Noda, M. (2007). Two distinct cytotoxic activities of subtilase cytotoxin produced by shiga-toxigenic Escherichia coli. Infect. Immun. 75, 488-496.

Morita, S., Kojima, T., and Kitamura, T. (2000). Plat-E: an efficient and stable system for transient packaging of retroviruses. Gene Ther. 7, 1063-1066.

Ogawa, M., Matsuda, R., Takada, N., Tomokiyo, M., Yamamoto, S., Shizukusihi, S., Yamaji, T., Yoshikawa, Y., Yoshida, M., Tanida, I., et al. (2018). Molecular mechanisms of Streptococcus pneumoniae-targeted autophagy via pneumolysin, Golgi-resident Rab41, and Nedd4-1-mediated K63-linked ubiquitination. Cell Microbiol. 20, e12846.

Sanjana, N.E., Shalem, O., and Zhang, F. (2014). Improved vectors and genome-wide libraries for CRISPR screening.

Nat. Methods 11, 783-784.

Shalem, O., Sanjana, N.E., Hartenian, E., Shi, X., Scott, D.A., Mikkelson, T., Heckl, D., Ebert, B.L., Root, D.E., Doench, J.G., and Zhang, F. (2014). Genome-scale CRISPR-Cas9 knockout screening in human cells. Science 343, 84-87.

Yahiro, K., Morinaga, N., Satoh, M., Matsuura, G., Tomonaga, T., Nomura, F., Moss, J., and Noda, M. (2006). Identification and characterization of receptors for vacuolating activity of subtilase cytotoxin. Mol. Microbiol. 62, 480-490.

Yahiro, K., Satoh, M., Morinaga, N., Tsutsuki, H., Ogura, K., Nagasawa, S., Nomura, F., Moss, J., and Noda, M. (2011) Identification of Subtilase Cytotoxin (SubAB) Receptors Whose Signaling, in Association with SubAB-Induced BiP Cleavage, Is Responsible for Apoptosis in HeLa Cells. Infect. Immun. 79, 617-627.

Yamaji, T., Nishikawa, K., and Hanada, K. (2010). Transmembrane BAX inhibitor motif containing (TMBIM) family proteins perturbs a trans-Golgi network enzyme, Gb3 synthase, and reduces Gb3 biosynthesis. J. Biol. Chem. 285, 35505-33518.

Yamaji, T., and Hanada, K. (2014). Establishment of HeLa cell mutants deficient in sphingolipid-related genes using TALENs. PLoS One 9, e88124.

Yamaji, T., Horie, A., Tachida, Y., Sakuma, C., Suzuki, Y., Kushi, Y., and Hanada, K. (2016). Role of Intracellular Lipid Logistics in the Preferential Usage of Very Long Chain-Ceramides in Glucosylceramide. Int. J. Mol. Sci. 17, E1761.

Yamaji, T., Sekizuka, T., Tachida, Y., Sakuma, C., Morimoto, K., Kuroda, M., and Hanada, K. (2019). A CRISPR Screen Identifies LAPTM4A and TM9SF Proteins as Glycolipid-regulating Factors. iScience 11, 409-424.
